# Microbial quality of agricultural water in Central Florida

**DOI:** 10.1371/journal.pone.0174889

**Published:** 2017-04-11

**Authors:** Zeynal Topalcengiz, Laura K. Strawn, Michelle D. Danyluk

**Affiliations:** 1 Department of Food Science and Human Nutrition, Citrus Research and Education Center, Institute of Food and Agricultural Sciences, University of Florida, Lake Alfred, Florida, United States of America; 2 Department of Food Science and Technology, Agricultural Research and Extension Center, Virginia Tech, 33446 Research drive, Painter, Virginia, United States of America; USDA-ARS Eastern Regional Research Center, UNITED STATES

## Abstract

The microbial quality of water that comes into the edible portion of produce is believed to directly relate to the safety of produce, and metrics describing indicator organisms are commonly used to ensure safety. The US FDA Produce Safety Rule (PSR) sets very specific microbiological water quality metrics for agricultural water that contacts the harvestable portion of produce. Validation of these metrics for agricultural water is essential for produce safety. Water samples (500 mL) from six agricultural ponds were collected during the 2012/2013 and 2013/2014 growing seasons (46 and 44 samples respectively, 540 from all ponds). Microbial indicator populations (total coliforms, generic *Escherichia coli*, and enterococci) were enumerated, environmental variables (temperature, pH, conductivity, redox potential, and turbidity) measured, and pathogen presence evaluated by PCR. *Salmonella* isolates were serotyped and analyzed by pulsed-field gel electrophoresis. Following rain events, coliforms increased up to 4.2 log MPN/100 mL. Populations of coliforms and enterococci ranged from 2 to 8 and 1 to 5 log MPN/100 mL, respectively. Microbial indicators did not correlate with environmental variables, except pH (*P*<0.0001). The *invA* gene (*Salmonella*) was detected in 26/540 (4.8%) samples, in all ponds and growing seasons, and 14 serotypes detected. Six STEC genes were detected in samples: *hly* (83.3%), *fliC* (51.8%), *eaeA* (17.4%), *rfbE* (17.4%), *stx-*I (32.6%), *stx-*II (9.4%). While all ponds met the PSR requirements, at least one virulence gene from *Salmonella* (*invA-*4.8%) or STEC (*stx-*I-32.6%, *stx-*II-9.4%) was detected in each pond. Water quality for tested agricultural ponds, below recommended standards, did not guarantee the absence of pathogens. Investigating the relationships among physicochemical attributes, environmental factors, indicator microorganisms, and pathogen presence allows researchers to have a greater understanding of contamination risks from agricultural surface waters in the field.

## Introduction

The United States Congress authorized the Food and Drug Administration (FDA) to set up minimum safety standards for the production and harvest of fruits and vegetables that may be consumed without any further processing [1]. Science based standards to minimize the risk of outbreaks related to the consumption of fruit and vegetables have been established in the Food Safety Modernization Act (FSMA) Final Rule on Produce Safety named as “Standards for the Growing, Harvesting, Packing, and Holding of Produce for Human Consumption; Final Rule” [2]. Agricultural water is considered one of the main microbial contamination risk routes for produce [2]. Agricultural water quality standards in the Produce Safety Rule evolved significantly between the proposed and final rule, published at the end of 2015.

In the proposed FSMA Produce Safety Rule in 2013, metrics for water quality allowed generic *Escherichia coli* populations in water that directly contacted the harvestable portion of the crop ≤ 235 MPN/100 mL, with a geometric mean (n = 5) ≤126 MPN/100 mL. Water exceeding this number was to be retested, not used, or used by reducing potential contamination risk of produce. A seven-day testing frequency was required for untreated surface water subjected to run off during the growing season [3].

On September 29, 2014, FDA published a supplemental notice that revised water quality requirements for agricultural waters under the proposed Produce Safety Rule. These water quality standards required the establishment of a “microbial water quality profile (MWQP)” for each surface water source by sampling 20 times as close as practical to harvest within a minimum of two years and calculating a geometric mean (GM) and a “statistical threshold value (STV)”. To meet the water quality standard, the geometric mean and STV should be ≤126 *E*. *coli* and ≤410 CFU *E*. *coli* in a 100 mL water sample, respectively. After establishing a MWQP, the GM and STV were annually calculated with the collection of new samples to determine if they supported the previously defined MWQP. If they do not support the previously defined MWQP, a new MWQP should have been established by combining most recent baseline or annual survey data and data from prior years to make up a data set of at least 20 samples [4].

The final Produce Safety Rule was published with revised agricultural water quality standards on November 13, 2015. In the final rule, development of a MWQP must be established with a baseline survey of a minimum of 20 surface water samples as close as practical to harvest, but limited to a minimum of two and a maximum of four years. GM and STV standards were kept the same as the supplemental notice. After the baseline survey, the MWQP of surface water must be verified with a minimum of five samplings as close as practical to harvest, annually. Generic *E*. *coli* populations from the five annual samples must be combined with the most recent 15 samples from the previous MWQP to calculate a rolling GM and STV instead of comparing to the previously established baseline. The new calculated GM and STV values should then each year be compared to the agricultural water standards for confirmation; the potential for corrective measures to be used in the case of water exceeding the MWQP were also introduced [2].

The microbial monitoring of agricultural surface waters is less frequent than for drinking and recreational waters. Generic *E*. *coli*, fecal streptococci, enterococci, and total coliforms are common indicator microorganisms for fecal contamination and microbial water quality [5]. The presence of pathogens, particularly *Salmonella* spp. and shiga-toxin producing *E*. *coli* (STEC), in agricultural water sources has been reported for different regions [6–16]. Rainfall events and agricultural practices that cause the distortion of sediments and run-off water often result in resuspension of microorganisms in agricultural surface water [17, 18]. The population of indicator microorganisms (such as generic *E*. *coli*) and pathogens (such as *E*. *coli* O157:H7 and *Salmonella* spp.) are affected differently depending on rainfall events in different regions and time of the year [5, 15, 19–22]. No strong correlation or clear trend between precipitation and microbial water quality were also reported in some studies [11, 17]. These studies raised questions about the effect of different amounts of precipitation, sampling times, and site locations that may influence the concentration of indicator microorganisms and pathogens. More data are needed regarding the populations of indicator microorganisms and pathogens in agricultural surface waters throughout the growing season and the effects of weather events on them.

The objectives of this study were to i) monitor indicator microbial populations in surface water weekly over the course of two growing seasons; ii) increase monitoring frequency during high water usage or weather events to determine their impact on microbial populations; iii) evaluate the final Produce Safety Rules agricultural water requirements for Central Florida surface water; iv) determine correlation between microbial indicators and physicochemical water attributes; and v) determine correlation of indicator organisms and the presence of *Salmonella* and STEC genes in the tested water samples. Data presented in here will help to understand if agricultural pond waters in Central Florida were in compliance with new Produce Safety Rule and if pathogens were present in compliant waters. This is the first study to evaluate microbial quality of ponds using the metrics in the final Produce Safety Rule.

## Materials and methods

### Water sampling

Six ponds within a four mile radius, in an area of West Central Florida with intensive specialty crop production, were selected for sampling. Permission to sample each pond was granted by the owner prior to commencement of the study. Ponds were in use for agricultural purposes, including those uses covered by the PSR, and were fed with well water. Physical conditions of the ponds are shown in Table 1. Surface water samples (500 mL) were collected with sterile glass bottles from ponds weekly, and every other day after rain events of 20 mm or more within 24 h, and after intensive surface water usage due to initial planting stage or frost protection. Surface water samples were collected during the dry season in Florida when irrigation of fields was essential; crops in all fields were irrigated by drip irrigation under plastic mulch. Sampling was performed prior to solar noon to minimize the influence of sunlight and to ensure a consistent time of sampling during the day. Samples were collected during three consecutive growing seasons from 2012 to 2015: 46 times in first year (2012–2013), 44 times in second year (2013–2014), and five times in the third year (2014–2015). In total 540 samples were collected during the 2012–2013 (276) and 2013–2014 (264) growing seasons from the six ponds. A third year (2014–2015 growing season) of water sampling was performed to simulate the annual testing required after a MWQP is achieved under the Produce Safety Rule; 5 water samples were collected from each pond for a total of 30 additional samples. GM and STV values, to establish MWQPs for each pond, were calculated using the excel tool developed by the Western Center for Food Safety, University of California, Davis (http://wcfs.ucdavis.edu/). Water (150 mL) from each sampling time and pond over the first two growing seasons was filtered through a sterile 0.45 μm pore-size filter (Millipore, Billerica, MA, USA) and the filters stored at -20°C for subsequent identification of *Salmonella* and STEC genes by PCR until all sampling was complete.

**Table 1:** Physical conditions of the ponds when sampling started in 2012.

Pond Code	Approximate Pond Age (years)	Approximate Pond Size (m^2^)	The conditions around pond	Sunlight exposure
Pond 1	3	5,100	Grassed, elevated, low run off	High
Pond 2	45–49*	3,850	Open soil, not elevated, high run off, connected to a creek	Low
Pond 3	3	7,250	Grassed, not elevated, low run off, tail water recovery	High
Pond 4	24	6,550	Grassed, not elevated, low run off	High
Pond 5	4–5	5,000	Grassed, not elevated, low run off	High
Pond 6	7	7,000	Lightly grassed, not elevated, medium run off	Medium

* Expanded 6 years ago.

### Microbiological analysis

Surface water samples were returned to the laboratory on ice immediately after sampling and microbial analysis of samples began within three hours. The populations of three different indicator microorganisms were enumerated by the most probable number method (MPN/100 mL) by IDEXX Quanti-Tray^®^ 2000 (IDEXX Laboratories, Westbrook, ME, USA). Quanti-Tray^®^ 2000/Colilert^®^ was used to examine total coliform and generic *E*. *coli* populations, and Quanti-Tray^®^ 2000/Enterolert^®^ for enterococci populations, respectively. Total coliform and *E*. *coli* populations were examined through the activity of β-galactosidase and β-glucuronidase, respectively. The activity of β-glucosidase was used for the enterococci population identification.

### Water attributes

Physicochemical attributes of water were tested on 100 mL samples for each pond with relevant instruments. Temperature of water, turbidity, conductivity, pH and oxidation-reduction potential (ORP) of samples were measured in triplicate. Turbidity was measured in Formazin Attenuation Units (FAU) with a portable colorimeter (DR/850, Hach Company, Loveland, CO, USA). Water temperatures (°C) were measured with a portable temperature probe (SH66A, Cooper Instrument Corporation, Middlefield, CT, USA). ORP (mV) was measured with a portable ORP meter (pH6 Acorn series, Oakton, 126 Vernon Hills, IL, USA). pH was measured with lab scale pH meter (Accumet^®^ AB15 Basic, Fischer Scientific, Pittsburg, PA, USA). Conductivity (μS/cm), was measured with a portable conductivity tester (HI98304 DIST^®^ 4 EC, HANNA Instruments, Woonsocket, RI, USA).

### *Salmonella* and STEC genes

For STEC, 25 mL of modified peptone water with pyruvate (mBPWp; Neogen, Lansing, MI, USA) was added to frozen filters, and incubated at 35 ± 1°C for 24 h. After preenrichment, DNA extraction was performed using MoBio UltraClean DNA isolation kit (MoBio, Carlsbad, CA, USA). For *Salmonella*, a subsequent enrichment was performed by addition of 0.1 mL of the preenrichment into 10 mL Rappaport-Vassilidas (RV) (Difco, Bectin, Dickinson, Sparks, MD, USA) medium and incubated at 42 ± 1°C for 48 h. DNA extraction was performed directly from RV with the same DNA extraction kit.

The presence of the *invA* gene was used to detect *Salmonella* spp. The *inv*A primer set protocol from Rahn et al. [23] was modified to use reagents Fisher *ex*ACTG*ene*™ PCR kit and Core Reagent Sets (Fisher Scientific, CA, USA). *S*. Braenderup H9812 and *E*. *coli* O157:H7: clinical isolate, spinach outbreak) were used as positive and negative controls, respectively. The PCR reagent concentrations for 50 μL reaction was as follows: 31 μL water, 10 μL 5x Go Taq Flexi buffer, 3 μL 25 mM MgCl2, 1 μL 10 mM dNTPs, 0.25 μL Go Taq DNA polymerase, and 2 μL 10 μM of each primer, and 1 μL DNA template. In practice, The PCR reaction mix was prepared with 20 μL water, 25 μL AmpliTaq Gold 360 (A&B Applied Biosystems, Foster City, CA, USA) that contains all reaction ingredients as listed above except for primers, 2 μL 10 μM of each primer and 1 μL DNA template. PCR conditions were: 10 min 94°C for melting, followed by 20 cycles of 94°C for 30 s, 62°C for 30 s, 72°C for 1 min, and with a final elongation of 72°C for 5 min. Gel electrophoresis was performed on 2.0% agarose gels with 1X TBE buffer (BIORAID, Hercules, CA, USA) at 120 V for 60 min. The gel was then placed in 400 mL of ethidium bromide (40 mg/mL) for 20 min for staining. Before UV light (MultiDoc-It Digital Imaging System; UVP, Upland, CA, USA) visualization, gels were destained in 400 mL deionized water twice for 20 min.

Extracted DNA was used in multiplex PCR assay targeting six STEC genes as previously described by Strawn et al. [15], Hu et al. [24], and Manuel [25]. The PCR assay was designed to amplify six genes including, *hly*, *fliC*, *eaeA*, *rfbE*, *stx-*I, and *stx-*II. STEC isolates (*E*. *coli* O111: clinical isolate, apple juice outbreak, NY and O157: clinical isolate, spinach outbreak) and *S*. Braenderup H9812 were used as positive and negative controls, respectively. For 50 μL reactions, 15.64 μL water, 25 μL AmpliTaq Gold 360 (A&B Applied Biosystems, Foster City, CA, USA) 8.36 μL primer mix and 1 μL DNA template were used. PCR conditions were: 10 min 95°C for melting, followed by 20 cycles of 94°C for 30 s, 59°C for 1 min with 0.5°C decreasing temperature per cycles, 72°C for 1 min, and a second 20 cycle of 94°C for 30 s, 49°C for 1 min, 72°C for 1 min and with a final elongation of 72°C for 7 min. Electrophoresis and staining were performed in the same way as in the protocol for the conventional PCR for *Salmonella* except that 130 V was used for 60 min during gel electrophoresis.

### *Salmonella* isolation and serotyping

Following PCR analysis, *Salmonella* positive samples were enriched by FDA Bacteriological Analytical Manual (BAM) to obtain a culture [26]. After RV enrichment, a loopful of *Salmonella* positive samples were streaked onto bismuth sulfite agar (BSA), xylose-lysine-desoxycholate (XLD), and hektoen enteric agar (BSA, XLD, HE; Difco, Becton, Dickinson, Sparks, MD, USA). XLD and HE were incubated at 35 ± 2°C for 24 h, and BSA was incubated at 35 ± 2°C for 48 h. Following incubation, if typical colonies were present, they were transferred into triple sugar iron agar (TSI; Difco, Becton, Dickinson, Sparks, MD, USA) and lysine iron agar (LIA; Difco, Becton, Dickinson, Sparks, MD, USA) slants. *Salmonella* isolates were frozen (-80°C) in 30% glycerol and 70% tryptic soy broth mixture (TSA; Difco, Becton, Dickinson, Sparks, MD, USA) for further confirmation tests. For serotyping, isolates were streaked onto xylose-lysine-tergitol-4 agar (XLT4; Difco, Bectin, Dickinson, Sparks, MD, USA) and incubated for 24 ± 2°C at 35 ± 2°C. All *Salmonella* isolates were also confirmed using a latex agglutination test (Oxoid, Hampshire, UK) before shipping isolates to the National Veterinary Services Laboratories (USDA, NVSL, Ames Iowa) for serotyping.

### Pulsed-field gel electrophoresis (PFGE)

Serotyped isolates were also analyzed for DNA fingerprinting using pulsed-field gel electrophoresis (PFGE). The standard *Salmonella* protocol, published by the Center for Disease Control and Prevention PulseNet, was performed for PFGE analysis [27]. Briefly, *Salmonella* Braenderup H9812 was used as the reference marker. DNA restriction was performed using the *XbaI* enzyme and fragments were separated by gel electrophoresis (CHEF- DRII system; Biorad, Hercules, California, USA). Following electrophoresis, the gel was stained and visualized under UV light (MultiDoc-It Imaging System; UVP, Upland, CA, USA). Gel images were analyzed with Bio-Numerics software 6.6 (Applied Maths; Sint-Martens-Latem, Belgium). PFGE profiles were evaluated for similarity using the unweighted pair group method with arithmetic mean algorithm (UPGMA) based on Dice coefficients with a maximum space tolerance of 1.5%.

### Rainfall data collection and animal activity observation

Rainfall data was collected from the Florida Automated Weather Network (FAWN) (http://fawn.ifas.ufl.edu/). The closest weather recording FAWN tower (Dover), within the 4-mile radius of the agricultural ponds was chosen and precipitation rates were followed daily during growing seasons. Animal activity in and around ponds was observed and noted based on the presence of animals and their tracks during sampling.

### Statistical analysis

Statistical analysis was achieved by analyzing data with JMP 11 software (SAS® Institute Inc., Cary, NC, USA 2013). All statistical analyses were performed on data collected only during 2012/2013 and 2013/2014 growing seasons (n = 540). Pearson product moment correlation coefficients (“r”) among the physicochemical characteristics of waters and indicator microorganisms were analyzed (*P* < 0.05). Linear regression of precipitation and microbial indicators was calculated by R-squared (R^2^). Data set used in the study are documented in S1 Table.

## Results

Water from six agricultural irrigation ponds was sampled for three consecutive years during growing seasons in Central Florida. A MWQP for the first year, a two-year baseline, and third year rolling MWQP consisting of GM and STV of generic *E*. *coli* were calculated for each pond, and are listed in Table 2. All agricultural ponds tested met the current FSMA Produce Safety Rule standards (GM≤126 *E*. *coli* and STV≤410 *E*. *coli* in 100 mL) for the MWQP 100.0% of the time after establishment of a baseline survey for two years. The GM values for all ponds were under the recommended limits. GM values ranged from 1 to 32 MPN/100 mL for all ponds, except for Pond 2 in the 2012/2013 growing season (GM = 118). STV values were lower than the set standards for all ponds during the two-year baseline and the third year rolling MWQPs. With the exception of Pond 2, all STV values were ≤303 and under Produce Safety Rule standards in the first season. The highest STV, from Pond 2 in the first season, was 5,571 and higher than the STV set limit (≤410).

**Table 2:** Calculated MWQP values for all ponds for three consecutive growing seasons (MPN/100 mL).

	First year		Two year baseline MWQP		Third year rolling MWQP	
Pond Code	GM	STV	GM	STV	GM	STV
Pond 1	7	94	1	2	1	4
Pond 2	118	5571*	8	103	11	137
Pond 3	3	19	2	6	2	4
Pond 4	14	`166	9	140	6	51
Pond 5	6	54	5	30	8	95
Pond 6	32	303	7	70	8	76

* Value that does not meet the produce safety rule criteria.

Turtles, fish, and birds were frequently and repeatedly observed in and around all ponds. Pond 2 had high animal activity during sampling including sightings or evidence of turtles, fish, frogs, alligators, and birds including ibis, heron, Sandhill cranes, wood storks, wild and farm ducks, and vultures.

### Microbial indicators

**Fig 1:**
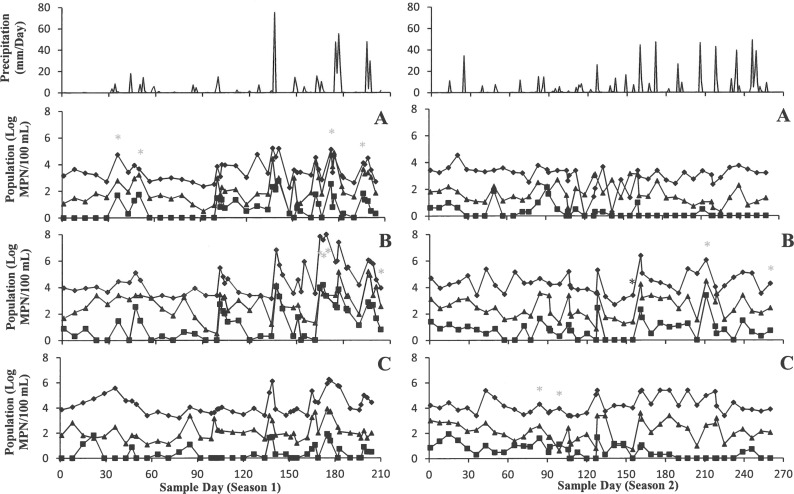
Most Probable Number of Total coliform (♦), Generic *E*. *coli* (■), and enterococci (▲) (Log MPN/100 mL) from six agricultural ponds (Pond 1: A, Pond 2: B, Pond 3: C) with daily rainfall (mm/Day) in Central Florida. *invA* positive sampling days were shown with “*”.

**Fig 2:**
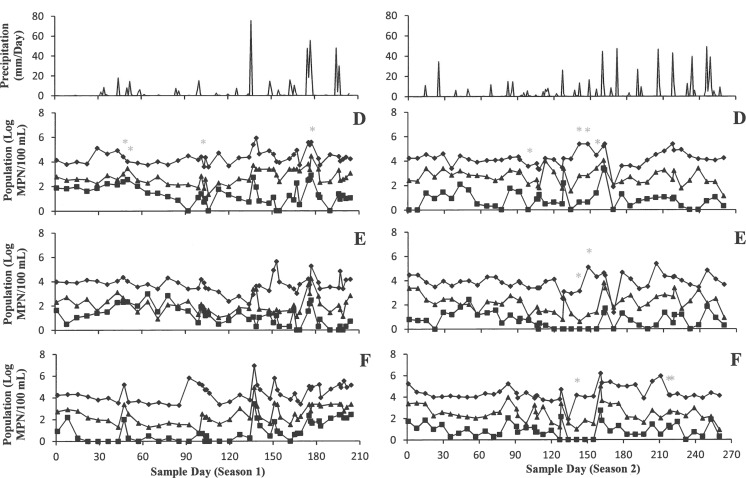
Most Probable Number of Total coliform (♦), Generic *E*. *coli* (■), and enterococci (▲) (Log MPN/100 mL) from six agricultural ponds (Pond 4: D, Pond 5: E, Pond 6: F) with daily rainfall (mm/Day) in Central Florida. *invA* positive sampling days were shown with “*”.

All microbial indicators including total coliform, enterococci, and generic *E*. *coli* followed similar trends (Figs 1 and 2). The population of indicators fluctuated during sampling time for 204 days in season 1 and 260 days in season 2. Total coliform populations ranged from 0 to 8 log MPN/100 mL and showed the highest populations in all ponds compared to enterococci and generic *E*. *coli*. Populations of enterococci were the second highest and ranged from 0 to 5.2 log MPN/100 mL. Populations of generic *E*. *coli* stayed well below the populations of the other two microbial indicators on average, and ranged from 0 to 4.2 log MPN/100 mL.

The amount of rain exceeded 20 mm within 24 h of sampling, 6 and 10 times during growing seasons 1 and 2, respectively. The maximum precipitation recorded was 75 mm within 24 h before sampling. Following rain events, total coliform populations increased up to 4.2 log MPN/100 mL from the prior sampling. Microbial populations within ponds varied up to 4 log MPN/100 mL between sample points. No strong correlations were calculated between precipitation and any microbial indicators in any pond. The highest correlations (R^2^) between rainfall and total coliforms were observed in Pond 1 and Pond 6, 0.15 and 0.40, respectively. Between rainfall and enterococci, the highest correlations (R^2^) were lower than other indicators and were observed in Pond 2 and Pond 6 were 0.11 and 0.22, respectively. Rainfall, 24 h (mm/Day) after sampling, did not correlate with generic *E*. *coli* populations for Pond 1, 3, 4, 5 (R^2^ < 0.1); however, generic *E*. *coli* populations correlated with rainfall the most in Pond 2 and Pond 6 with R^2^ values of 0.16 and 0.42, respectively. There were no other major correlations between factors and indicators, as R^2^ values were below 0.1 (Table 3).

**Table 3:** Precipitation and microbial indicator correlations for all ponds.

Pond Code	Precipitation Correlation (R^2^)		
	Total Coliform	Enterococci	Generic *E*. *coli*
Pond 1	0.1468	0.0631	0.0335
Pond 2	0.0026	0.1090	0.1586
Pond 3	0.0094	0.0406	0.0113
Pond 4	0.0392	0.0264	0.0195
Pond 5	0.0000	0.0150	0.0021
Pond 6	0.3956	0.2261	0.4242

### *Salmonella* and STEC gene presence

At least one *Salmonella* or STEC gene was detected in 91.3% of samples. All STEC genes tested (n = 6) were detected in only 2.6% of the samples, concurrently. Average individual STEC gene detected in all ponds was as the following percentages: *stx-*I-32.6%, *stx-*II-9.4%, *eaeA*-17.4%, *hly*-83.3%, *rfbE*-17.4%, *fliC-*51.8%. The individual percentage of each STEC gene in the different ponds varied (Table 4) including: *stx-*I: 28.9–35.6%, *stx-*II: 7.8–13.3%, *eaeA*: 12.2–24.4%, *hly*: 80–86.7%, *rfbE*: 13.3–22.2%, *fliC*: 50–54.4%. The *invA* gene (*Salmonella*) was detected in 26/540 (4.8%) of samples. The *invA* gene was detected in all ponds and the percentages ranged from 2.2 to 8.9% (Table 4). Interestingly, 57.7% (15/26) of the *invA* positive samples were from Ponds 2 and 4.

**Table 4:** The positive percentages of STEC and *invA* genes (%) for each pond.

Pond Code	Positive STEC Genes						All STEC	
	*stx-*I	*stx-*II	*eaeA*	*hly*	*rfbE*	*fliC*		*invA*
Pond 1	32.2	7.8	20	83.3	22.2	52.2	0.2	4.4
Pond 2	35.6	7.8	16.7	82.2	13.3	50	0.4	7.8
Pond 3	35.6	10	24.4	80	22.2	52.2	0.6	2.2
Pond 4	32.2	8.9	12.2	84.4	15.6	52.2	0.0	8.9
Pond 5	28.9	8.9	17.8	86.7	16.7	54.4	0.9	2.2
Pond 6	31.1	13.3	13.3	83.3	14.4	54.4	0.6	3.3

### *Salmonella* serotyping

Only 21 of 26 *Salmonella* isolates were sent to the National Veterinary Science Lab (NVSL; Ames, IA) for serotyping. Isolates from the other five samples were not obtained due to technical difficulties in the laboratory (i.e. isolates not recovered from frozen storage). Fourteen different serotypes were detected (Table 5). *Salmonella* Saintpaul and *Salmonella* 4, 12,:i:- were isolated two and three times from separate ponds, respectively. *Salmonella* 4, 5, 12:i:- was isolated four times in three different ponds. Other serotypes isolates from ponds included Anatum, Anatum_var._15+, Baildon, Florida, Hartford, Javiana, Muenchen, Newport, Rough O:i:-, IV_50:z4,z23:- and Rubislaw.

**Table 5:** List of *Salmonella* isolates and summary of tests applied to each *invA* positive sample.

Sample information			From RV	Isolation and Confirmation tests			NVSL, USDA*
Isolate code	Pond code	Sample date	PCR (*invA*)	BAM	XLT4	Latex	Serotype
SA4	Pond 4	12/23/2012	**+**	N/A^#^	N/A^#^	N/A^#^	N/A^#^
SA32	Pond 2	04/22/2013	**+**	N/A^#^	N/A^#^	N/A^#^	N/A^#^
SA40	Pond 4	05/02/2013	**+**	N/A^#^	N/A^#^	N/A^#^	N/A^#^
SA55	Pond 1	12/12/2012	**+**	**+**	**+**	**+**	Saintpaul
SA70	Pond 4	02/15/2013	**+**	**+**	**+**	**+**	4, 5, 12:i:-
SA80	Pond 2	04/20/2013	**+**	**+**	**+**	**+**	Saintpaul
SA86	Pond 2	04/24/2013	**+**	**+**	**+**	**+**	Anatum_var._15+
SA92	Pond 2	05/24/2013	**+**	**+**	**+**	**+**	Hartford
SA103	Pond 1	12/26/2012	**+**	**+**	**+**	**+**	4, 12:i:-
SA106	Pond 4	12/26/2012	**+**	**+**	**+**	**+**	4, 12:i:-
SA133	Pond 1	04/30/2013	**+**	**+**	**+**	**+**	Baildon
SA139	Pond 1	05/21/2013	**+**	**+**	**+**	**-**	Javiana
SB38	Pond 2	05/07/2014	**+**	N/A^#^	N/A^#^	N/A^#^	N/A^#^
SB63	Pond 3	12/30/2013	**+**	**+**	**+**	**+**	4, 5, 12:i:-
SB76	Pond 4	03/09/2014	**+**	**+**	**+**	**+**	4, 5, 12:i:-
SB77	Pond 5	03/09/2014	**+**	**+**	**+**	**+**	4, 12:i:-
SB78	Pond 6	03/09/2014	**+**	**+**	**+**	**+**	Anatum
SB90	Pond 6	05/15/2014	**+**	**+**	**+**	**+**	Newport
SB92	Pond 2	06/04/2014	**+**	**+**	**+**	**+**	Muenchen
SB111	Pond 3	01/15/2014	**+**	**+**	**+**	**+**	Rough_O:i:-
SB112	Pond 4	01/15/2014	**+**	**-**	N/A^#^	N/A^#^	N/A^#^
SB138	Pond 6	05/14/2014	**+**	**+**	**+**	**-**	Florida
SB218	Pond 2	03/12/2014	**+**	**+**	**+**	**-**	IV_50:z4,z23:-
SB220	Pond 4	03/12/2014	**+**	**+**	**+**	**+**	4, 5, 12:i:-
SB244	Pond 4	02/26/2014	**+**	**+**	**+**	**+**	Rubislaw
SB245	Pond 5	02/26/2014	**+**	**+**	**+**	**+**	4, 5, 12:i:-

* NVSL, USDA: National Veterinary Services Laboratories, US Department of Agriculture.

^#^ N/A: Not applicable, no isolate was obtained.

### Water attributes and microbial indicators

Physicochemical characteristics for all six agricultural irrigation ponds were plotted for the first two growing seasons. Water and air temperature, pH and ORP, turbidity and conductivity plots are displayed in supplemental S1–S3 Figs. Multiple linear regression analysis with populations of total coliform, enterococci and generic *E*. *coli*, air temperature, water temperature, conductivity, pH, oxidation reduction potential, and turbidity indicated that all microbial indicators correlated with conductivity, pH, and turbidity (*P* < 0.05). Pearson product moment correlation coefficients (r) and *P* values for all attributes are listed in Table 6. Correlation coefficients demonstrate stronger positive and negative linear relationships when they are closer to 1 and -1. The correlations between microbial indicators and water attributes were below r < 0.2 except for pH. Correlation coefficients among microbial indicators were above 0.58 (*P* < 0.0001). The highest correlation coefficient among the microbial indicators, 0.63, was calculated between generic *E*. *coli* and enterococci.

**Table 6:** Pearson product moment correlation coefficients (r) with *P*-values determined between each of the physical, chemical, and biological water attributes for all ponds combined.

Water Attributes	*E*. *coli*	Enterococci	Air temperature (°C)	Water temperature (°C)	Conductivity (μS/cm)	pH	ORP (mV)	Turbidity (FAU)
Coliforms	0.5868* (<0.0001)	0.5787* (<0.0001)	0.0905* (0.0365)	0.1563* (0.0003)	0.1191* (0.0058)	-0.2094* (<0.0001)	-0.0672 (0.1209)	0.0851* (0.0495)
*E*. *coli*		0.6328* (<0.0001)	-0.0492 (0.2561)	-0.0004 (0.9919)	0.1805* (<0.0001)	-0.1983* (<0.0001)	-0.1170* (0.0068)	0.2187* (<0.0001)
Enterococci			-0.0248 (0.5675)	0.1011* (0.0195)	0.1049* (0.0153)	-0.2104* (<0.0001)	-0.1027* (0.0176)	0.2880* (<0.0001)
Air temperature (°C)				0.7449* (<0.0001)	-0.1537* (0.0004)	0.3286* (<0.0001)	-0.0158 (0.7154)	0.0344 (0.4284)
Water temperature (°C)					-0.1568* (0.0003)	0.2484* (<0.0001)	-0.0597 (0.1682)	0.0028 (0.9494)
Conductivity (μS/cm)						0.0564 (0.1929)	-0.1949* (<0.0001)	0.1050 (0.0153)
pH							-0.1727* (<0.0001)	0.1063* (0.0141)
ORP (mV)								-0.0424 (0.3284)

* Correlation coefficients are statistically significant (*P* < 0.05).

### PFGE profile analysis of serotyped *Salmonella* isolates

**Fig 3:**
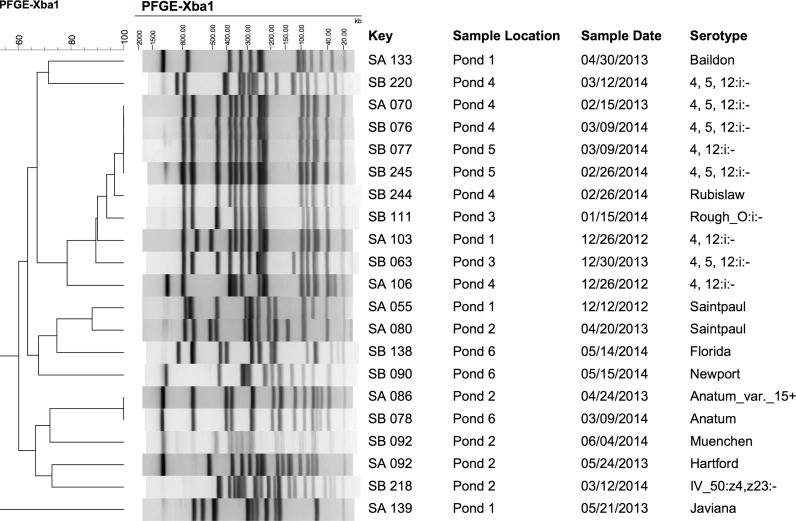
*XbaI* PFGE patterns for the serotyped *Salmonella* isolates (n = 21) collected from the six agricultural ponds in Central Florida during the two-year survey. Band sizes (kb) are displayed at the top of the PFGE pattern images. PFGE pattern order displayed is result of BioNumerics similarity analyses using the unweighted pair group-matching algorithm (UPGMA) and the Dice correlation coefficient with a maximum space tolerance of 1.5%. Key represents the isolate designation, sample location the pond number, sample date the month/year of sample collection, and serotype.

A dendrogram of all PFGE profiles showed that there was a high diversity of *Salmonella* from Central Florida surface waters (Fig 3). There were 17 different PFGE patterns for the 21 *Salmonella* isolates with only a few cases of identical PFGE patterns. Evidence of repeated *Salmonella* isolation was observed as indicated by an identical PFGE pattern over multiple samplings. For instance, the same PFGE pattern (serotype *S*. 4, 5, 12:i:- and *S*. 4,12:-:-) was found in Pond 4 and 5 during multiple sampling visits (Fig 3). Additionally, the same PFGE pattern (serotype *S*. Anatum and *S*. Anatum var 15+) was found in two different ponds (Ponds 2 and 6) during separate growing seasons (Fig 3).

## Discussion

The experimental design of this study was planned based on the proposed Produce Safety Rule in 2013 that required a seven-day testing frequency for untreated surface water subjected to run off [3]; we intended to evaluate the efficiency of the proposed rule for Central Florida surface waters by monitoring generic *E*. *coli* populations of chosen ponds weekly for two consecutive growing seasons. However, the supplemental notice published in 2014 significantly changed the requirements for surface water testing, resulting in the addition of a third year of water testing to support MWQPs (established in Y1 and Y2 of the study) [4]. The Produce Safety Final Rule was published in November 2015, and again changed surface agricultural water requirements. The Final Rule requires a baseline survey to be developed within two to four years, followed by five annual samplings to combine with the most recent 15 samplings to calculate a new rolling MWQP profile for each subsequent year [2]. The data collected here were able to fulfill the Final Rule agricultural water requirements, as the two-year baseline MWQPs were already calculated for each pond, and a third year sampling was performed to calculate a third year rolling MWQP.

Although only generic *E*. *coli* is considered a fecal contamination indicator and marker for microbial water quality in the Produce Safety Rule [2]; other indicators such as fecal streptococci, enterococci, and total coliforms are also commonly used [5]. Pachepsky et al. [28] summarized experimental data about the relationships between thermotolerant coliform, pathogens, and generic *E*. *coli* that proposed relationships were significant in 35% of all instances in surface waters suitable for irrigation. In this study, three types of microbial indicators including total coliform, enterococci, and generic *E*. *coli* were monitored to gain a better understanding of correlations and how populations of microbial indicators behaved. Microbial indicators tested in this study yielded moderate Pearson product moment correlation coefficients correlations (r) ranging 0.58 to 0.63 (Table 6). This situation suggests the use of generic *E*. *coli* or other indicator microorganisms tested as fecal contamination will yield acceptable results for surface waters in Central Florida. In a recent surface water survey study in Pennsylvania, a high correlation was calculated between total coliform and generic *E*. *coli* (r = 0.918), but lower correlations were observed with enterococci and total coliform (r = 0.362) and *E*. *coli* (r = 0.380) [6].

In this study, all microbial indicators correlated with each other moderately (r ≥ 0.58), but poorly with physicochemical water attributes. When all the water attribute data from the six ponds were pooled for analysis (n = 540 for each attribute), none of these attributes led to correlations in the acceptable range to predict the indicator populations (Table 6). Six microbial indicators monitored in Pennsylvania were not predicted with seven physical and environmental characteristics, similar to those evaluated here [6]. Similar to our findings, the highest correlation with all microbial indicators was pH (r > 0.2) and the occurrence of *E*. *coli* O157:H7 was negatively correlated with ORP as in previous reports [6, 7]. The positive conductivity correlation with microbial indicators in this study conflicted with previously reported inverse correlations in estuary waters in North Carolina and surface waters in Central Florida possibly due to changes in salinity and other dissolved solids, and dilution of *E*. *coli* concentrations in waters after storm and run-off water exposure [11, 29, 30]. This might be explained in that the pond waters in this study are close-system surface water sources feed with well water, and that the length of the sampling period was longer, more frequent, and concentrated on only six ponds. McEgan et al. [11] and Gonzales et al. [29] sampled a total of 202 and 151 times, respectively, compared to 540 data points in this study. In a study conducted in South Africa, total and fecal coliforms did not correlate with chemical oxygen demand; enterococci correlated with pH, turbidity, and rainfall [31].

Precipitation and agricultural activities can cause contamination of agricultural surface water and an increase in concentration of indicator microorganisms [18]. None of our indicator populations showed consistent relationships with precipitation rates. Rainfall occasionally influenced the indicator populations; a limited number of increases in indicator concentration from specific ponds was observed after high precipitation rates. This might be explained by the physical conditions of each pond and their run-off exposure probability. The highest indicator populations after rain were monitored in Pond 2 which is over 40 years old with open soil edges, a high run-off possibility, not elevated and connected to a creek. The highest precipitation and microbial indicator correlations were obtained in Pond 6 for total coliform, enterococci and generic *E*. *coli*. No particular reason was observed for the exceptional results of Pond 6; however, strong effect of sampling site difference was witnessed on microbial indicator populations.

Inconsistent results have been reported previously in studies evaluating correlations between precipitation and microbial populations including pathogens. In California, generic *E*. *coli* and *E*. *coli* O157:H7 population increase correlated with flow rate increase in rivers after rainfall [19]. *Salmonella* Agona isolates correlated significantly with rainfall in marine environment conducted in shellfish production areas of Galicia in Spain [20]. Santiago-Rodriguez et al. [21] reported that the concentration of thermotolerant coliforms decreased after 10.2 mm of rain 24 h prior to sample collection owing to possible dilution effect and increased after >25.4 mm of rain 48 h prior to sample collection due to possible sediment distortion. In a recent study, the average rainfall from previous day, week, and month prior to sampling did not significantly correlate with bacterial levels in Central Florida Surface waters [11]. In Georgia, *E*. *coli* O157:H7 and *Salmonella* positively correlated with precipitation levels [7, 8]. Won et al. [22] reported no association between microbial indicators and precipitation in reservoirs in Ohio; however, total coliform and generic *E*. *coli* counts in canals were higher after heavy precipitation (>20 mm) compared to lighter rainfall amounts. All microbial indicators tested here showed similar fluctuations in population after weather events consistent with calculated statistical correlations among them (0.63 ≥ r ≥ 0.58). Different results suggest that there might be some correlation on microbial population and precipitation in surface waters, whereas degree and type of effect might change based on other characteristics of water source. In this study, correlation variability in six different agricultural ponds in a four mile radius area indicates unreliability of the use of only rainfall data in the prediction of precipitation effect on microbial water quality. Instead, microbial water quality, physical conditions of water source, and run-off exposure assessment should be considered for prediction and risk assessment.

The presence of pathogens in surface water is expected due to possible exposure to animals, humans, weather and other environmental factors [18]. *Salmonella* can be found in any surface water, particularly in the Southeastern U.S. where they have been reported in different types of surface water sources at the overall frequencies between 37 to 100% in water samples sizes ranging from 1 mL direct MPN enrichment to 10 L tangential flow filtration followed by enrichment in North Carolina, Georgia, Central and North Florida [8, 10, 11, 13, 14]. McEgan et al. [11] reported that *Salmonella* can be found in Central Florida surface water throughout the year similar to other water survey studies conducted in the Southeastern U.S. [8, 10, 13, 14]. Here, the *invA* gene was detected in all ponds and both growing seasons, indicating the presence of *Salmonella* in agricultural pond waters in Central Florida is similar to other surface water in the Southeast U.S. However, the distribution of detected *invA* positive samples was not equal for each pond, and more than half of the positive samples were obtained from ponds 2 and 4, where the MWQPs, while under required standards, were the highest.

The number of studies evaluating prevalence of STEC in irrigation water in the Southeast region of the U.S. is limited compared to *Salmonella*. Gu et al. [7] reported that all 10 irrigation ponds sampled as three 500 mL aliquots of each water sample in monthly sampling from Suwannee River Watershed in Georgia were positive for *E*. *coli* O157:H7 some of the time during sampling period. Several water survey studies in other regions have showed the presence of STEC in agricultural water. The prevalence of *E*. *coli* O157:H7 was 0.9% and 2% in 90 mL water samples in two independent surveys about surface water in Sothern Alberta, Canada [9, 32]. In Netherlands, *E*. *coli* O157:H7 was found in 100 and 1000 mL of private water supply samples 2.7% of private wells [33]. In Argentina, surface water samples in 48 cattle feedlots yielded 70.5% *rfb* positive and 12.7% STEC in runoff from corrals exposed surface waters and 60.0% *rfb* positive and 10.0% STEC in non-exposed surface waters sampled as two liters with Moore swabs [34]. In this study, all six genes were detected in different percentages. The percentage of all these STEC genes were similar in all ponds, with hemolysin (*hly*), H7 (*Flic*), and *stx-*I being the most abundantly detected and *stx-*II being the least detected.

The correlations between microbial indicators and the presence of pathogens were concluded as possible in previous studies with similar findings, whereas this might be temporal, unsystematic or place and time specific. Ijabadeniyi et al. [31] reported high correlations between total and fecal coliforms and the presence of *Salmonella* in sampling sites including canal and two rivers in South Africa. However, generic *E*. *coli* population did not significantly associate with the presence of either *E*. *coli* O157 or *Salmonella* in water or sediment samples in a study done along the Central Coast of California [35]. Generic *E*. *coli* and enterococci did not correlate with *stx* gene encoded by STEC in recreational waters in Pennsylvania and water bodies in Central California [36, 37]. Fecal indicators correlated with *Salmonella*, significantly in the same study [37]. Higher correlations were observed at sites where indicator microorganisms and pathogens are detected in high populations for extended period of time [38]. Surface waters tested met the Produce Safety Rule standards for microbial water quality; however, at least one virulence gene from *Salmonella* (*invA-*4.8%) or STEC (*stx-*I-32.6%, *stx-*II-9.4%) was detected. This suggests the potential presence of pathogens in Central Florida surface agricultural water that meet Produce Safety Rule standards. During a risk assessment of microbial acceptability of agricultural water, the MWQP based on indicator organisms might not be enough to represent the safety of surface waters.

The source of pathogens in these ponds are potentially wild and domesticated animals, naturally found pathogens in water and sediment, and carried pathogens via runoff water from lands around ponds [18]. The sampled ponds, except for Pond 2, are small bodies of water with no connection to other natural water sources. Turtles, fishes, frogs, alligators, and various types of bird including ibis, heron, Sandhill cranes, wood storks, wild and farm ducks, and vultures were observed in and around the ponds. It has been reported that reptiles, wild and domesticated animals, household animals, and birds are some of the biotic sources of *Salmonella* [39–41]. Turtles were almost always observed in the ponds with high *invA* detection. Gaertner et al. [39] isolated *Salmonella* from wild turtles, but not from the corresponding water samples. This suggests that the presence of *Salmonella* in turtles is not enough to predict *Salmonella* in corresponding water; *Salmonella* is naturally harbored in healthy turtle intestines, and defecation can increase the potential risk of contamination [42].

Identical PFGE patterns were observed for *Salmonella* isolates from different and the same ponds, as well as in different growing seasons. Cases where isolates share the same PFGE pattern in the same pond over two consecutive growing seasons may indicate the survival of *Salmonella* for over a year despite unstable environmental conditions, or the re-introduction of *Salmonella* from a source. Cases where isolates share the same PFGE pattern in different ponds may suggest the possibility of a common source that moved between the ponds. For instance, the same PFGE pattern was found in Pond 6 and 2 and may be due to the same source or carrier, probably avian, since these ponds were in close proximity (all ponds within a four-mile radius) and had evidence of high wildlife. The presence of 17 different PFGE patterns from the 21 *Salmonella* isolates analyzed by PFGE demonstrates a high diversity of *Salmonella* in agricultural ponds from Central Florida. Previously, PFGE patterns from 221 *Salmonella* isolates obtained from Central Florida surface waters generated 11 major genotypic clusters with high diversity and identical banding pattern in the samples from different sampling seasons and sources [43]. High diversity and repeat isolation of identical PFGE patterns in different sources over time were found in different regions including, South Florida and New York State [15, 16].

The microbial quality of water that comes into contact with the harvestable portion of produce is commonly believed to directly relate to the safety of produce, and metrics describing indicator organisms are commonly used in the produce industry to ensure safety of a product [1]. The Produce Safety Rule requires growers to develop a MWQP for their agricultural water source based on the population of generic *E*. *coli*. Developed MWQPs for all ponds tested in Central Florida indicate that each pond met the requirements for microbial water quality set by FSMA; however, this does not guarantee the safety of these agricultural waters. Several times, populations of generic *E*. *coli* passed the previously proposed water quality recommendations by FDA where a single sample could not exceed 235 MPN/100 mL of generic *E*. *coli* and the geometric mean (n = 5) could not surpass 126 MPN/100 mL [3]. The Leafy Greens Marketing Agreement [44] still uses these recommendations, and the Florida Tomato Good Agricultural Practices standard only allows the use of potable water for fruit contact [45]. The Produce Safety Rule allows growers to use water exceeding these standards under some conditions. How the microbiological quality of surface water relates to, and should be evaluated, in regard to produce safety requires further evaluation. Understanding the relationships between indicator microorganisms, the presence of pathogens, and physicochemical attributes of water allows for a greater understanding of agricultural water and the risks it may pose to produce safety.

## Supporting information

S1 Table Data set used in the study. (PDF)

S1 Fig Air temperature (●) and Water temperature (▲) from six agricultural ponds (Pond 1: A, Pond 2: B, Pond 3: C, Pond 4: D, Pond 5: E, Pond 6: F) during 2012–2013 (46 data points) and 2013–2014 (44 data points) growing season in Central Florida. (TIF)

S2 Fig pH (●) and ORP (mV) (▲) from six agricultural ponds (Pond 1: A, Pond 2: B, Pond 3: C, Pond 4: D, Pond 5: E, Pond 6: F) during 2012–2013 (46 data points) and 2013–2014 (44 data points) growing season in Central Florida. (TIF)

S3 Fig Conductivity (μS/cm) (●) and Turbidity (FAU) (▲) from six agricultural ponds (Pond 1: A, Pond 2: B, Pond 3: C, Pond 4: D, Pond 5: E, Pond 6: F) during 2012–2013 (46 data points) and 2013–2014 (44 data points) growing season in Central Florida. (TIF)
