# Supplementary material for: Microbial quality of agricultural water in Central Florida
Source: PLoS One. 2017 Apr 11;12(4):e0174889. doi: 10.1371/journal.pone.0174889 (PMC5388333; doi:10.1371/journal.pone.0174889)
Supplement: S1 Table — (PDF) [file pone.0174889.s001.pdf]

Zeynal Topalcengiz
Laura K. Strawn
Michelle D. Danyluk
Microbial Quality of Agricultural Water in Central Florida

S1 Table. Data set used in the study.

			MPN/100 mL				Chemical and Physical Analysis				
Pond	Date	Sampling Time	Total Coliform	*E. coli*	Enterococci	Temp °C (air)	Temp °C (water)	Conductivity (μS)	pH	ORP(mV)	Turbidity(FAU)
1	11/7/2012	11:10 AM	1460	1	12.1	17.7	20.6	186	9.81	110	24
1	11/14/2012	11:20 AM	4480	1	31.3	23.2	21.4	208	10.17	102	31
1	11/21/2012	9:13 AM	2310	1	17.3	20.7	18.6	200	9.6	119	21
1	11/28/2012	11:21 AM	1732.9	1	70.6	25.4	19.9	217	9.96	101	35
1	12/5/2012	11:05 AM	2419.6	1	35.4	29	21.1	220	9.97	105	69
1*	12/12/2012	9:20 AM	54750	50.4	2419.6	22.1	22.2	232	8.65	111	24
1	12/19/2012	9:22 AM	2650	2	90.9	20.8	19.1	231	9.29	110	18
1	12/23/2012	12:08 PM	8620	19.7	920.8	17.6	17.8	252	9.18	114	19
1*	12/26/2012	11:25 AM	4650	55.6	1732.9	24.1	19.2	249	9.48	113	40
1	1/2/2013	11:29 AM	547.5	1	30.1	27.3	20.6	252	9.54	157	39
1	1/9/2013	9:56 AM	816.4	1	49.7	26.7	21.8	253	9.8	97	74
1	1/16/2013	9:43 AM	980.4	1	26.9	24.6	21.6	250	9.99	71	13
1	1/23/2013	9:43 AM	770.1	1	49.5	15.9	16.8	276	9.05	62	22
1	1/30.2013	9:37 AM	461.1	1	9.7	23.3	21.2	268	9.41	36	33
1	2/6/2013	9:51 AM	224.7	1	3.1	20	17.8	265	9.45	82	29
1	2/13/2013	9:50 AM	325.5	1	8.5	23.6	17.8	753	8.69	80	47
1	2/15/2013	10:04 AM	7030	29.8	2419.6	13.1	18.2	336	9.52	109	21
1	2/17/2013	9:40 AM	1203.3	6.3	58.6	9.2	16.5	272	9.29	41	27
1	2/18/2013	10:09 AM	9590	22.8	193.5	15.5	18.6	298	8.65	136	16
1	2/20/2013	9:42 AM	8820	5.2	98.5	19.9	19.1	291	8.72	223	17
1	2/27/2013	9:41 AM	8130	22.1	133.3	19.6	22	294	9.16	109	13
1	3/6/2013	9:58 AM	1046.2	3.1	9.7	17.4	18.2	289	9.19	123	24
1	3/13/2013	9:55 AM	57940	7.4	63.1	16.9	18.2	273	9.17	115	16
1	3/20/2013	9:15 AM	2160	4.1	60.2	19.8	21.5	269	9.29	109	15
1	3/23/2013	7:32 AM	166400	209.8	27550	18	18.8	250	9.16	58	26
1	3/25/2013	9:39 AM	36540	127.4	325.5	19.2	20.2	250	8.88	68	17
1	3/27/2013	9:40 AM	156500	579.4	980.4	9.4	17	263	8.64	10	46
1	4/3/2013	10:11 AM	172.3	2	2	24.3	23.5	251	9.94	60	20
1	4/6/2013	9:35 AM	3840	1	980.4	17.2	20.6	249	9.48	76	20
1	4/8/2013	10:00 AM	2419.6	5.2	13.4	23.8	22.5	249	9.51	90	15
1	4/10/2013	9:50 AM	2560	3.1	12.1	23.9	22.9	251	9.41	93	20
1	4/17/2013	9:13 AM	1553.1	1	75.9	24.4	25.8	270	9.17	82	28
1	4/20/2013	9:38 AM	32550	55.7	16160	19.6	25.4	259	8.91	75	29
1	4/22/2013	9:27 AM	4080	10.7	1046.2	19.3	24.1	263	8.57	80	28
1	4/24/2013	9:57 AM	686.7	1	60.5	23.8	24.4	266	8.64	94	16
1*	4/30/2013	10:08 AM	131700	344.8	51720	23	25.4	259	8.46	85	26
1	5/1/2013	9:51 AM	2419.6	6.2	7120	23.2	25.8	274	8.12	104	17
1	5/2/2013	8:54 AM	41060	39.5	81640	20.7	24.2	260	7.94	57	24
1	5/7/2013	9:45 AM	1413.6	1	227.9	20.3	24.3	257	8.55	55	30
1	5/8/2013	9:42 AM	980.4	2	74.3	24.4	24.6	256	8.7	48	31
1	5/15/2013	9:42 AM	410.6	1	36.7	24.7	25.5	262	8.83	35	20
1*	5/21/2013	9:45 AM	12340	65.7	4480	25.5	27.9	245	8.92	57	28
1	5/22/2013	9:42 AM	9600	21.6	1986.3	24.7	27.3	246	8.81	1	21
1	5/24/2013	9:43 AM	29090	17.5	2419.6	28.4	27.6	204	8.08	41	22
1	5/26/2013	9:39 AM	3730	3.1	1119.9	26.7	26.6	205	8.32	72	17
1	5/29/2013	9:45 AM	488.4	2	71.4	25.6	26.9	204	9.08	94	14
1	10/9/2013	9:00 AM	2620	4.1	63.8	22.9	26.9	158	7.82	82	14
1	10/16/2013	9:10 AM	1732.9	4.1	71.2	22.8	26	85	7.86	93	5
1	10/23/2013	9:00 AM	4110	9.6	154.1	24.1	27.7	228	7.69	117	9
1	10/30/2013	8:50 AM	34410	4.1	65.2	21.5	23.4	298	8.46	74	1
1	11/6/2013	9:00 AM	2920	1	17.2	24.6	23.3	330	8.73	26	7
1	11/13/2013	9:00 AM	2419.6	1	12.1	17.7	21.4	320	9.08	37	18
1	11/20/2013	9:15 AM	1986.3	1	10.9	24	22.7	260	8.58	77	8
1	11/27/2013	9:10 AM	2560	72.3	161.6	20.2	21.3	254	8.65	11	9
1	12/3/2013	9:30 AM	3740	1	13.4	22	19.7	271	8.72	39	8
1	12/11/2013	9:30 AM	1732.9	1	28.5	19.8	22.1	170	8.7	73	9
1	12/18/2013	9:15 AM	1553.1	2	15.2	13	18	124	8.68	87	9
1	12/23/2013	9:15 AM	325.5	2	27.5	24.1	21.5	179	9.05	53	8
1	12/30/2013	9:00 AM	5880	9.7	307.6	17	18.8	113	8.84	84	18
1	1/7/2014	8:50 AM	2880	135.4	161.6	2.8	18.5	19.5	7.35	147	13
1	1/8/2014	9:00 AM	1732.9	42.6	59.2	7.5	16.7	175	7.38	129	10
1	1/15/2014	9:20 AM	2419.6	3.1	488.4	12.5	17.5	154	7.46	95	10
1	1/22/2014	9:20 AM	2419.6	1	35	7	13.7	168	7.34	131	5
1	1/23/2014	9:55 AM	410.6	2	4.1	9.5	14.1	130	7.03	132	4
1	1/24/2014	9:20 AM	1046.2	1	1	8.6	12	130	7.18	154	6
1	1/29/2014	9:00 AM	2419.6	3.1	110.6	13.6	16.5	155	7.35	119	9

1	2/5/2014	9:40 AM	1	1	37.2	23.9	20.7	141	7.04	192	11
1	2/12/2014	9:15 AM	26.6	1	1046.2	17.4	17.9	154	7.18	179	10
1	2/13/2014	10:00 AM	111.9	2	1299.7	14.9	17.5	124	6.93	96	7
1	2/19/2014	9:00 AM	4640	2	50.5	19.3	18.1	138	7.38	123	12
1	2/26/2014	9:15 AM	2	1	478.6	16.8	21.1	161	8.09	195	11
1	3/5/2014	9:30 AM	488.4	1	23.7	18.5	21.5	154	8	164	10
1	3/12/2014	9:00 AM	23.1	1	1413.6	20.5	21.3	227	8.85	137	23
1	3/18/2014	9:00 AM	2419.6	9.7	1299.7	16.5	21.1	136	8.86	106	48
1	3/19/2014	9:05 AM	816.4	1	32	16.5	21.2	140	8.39	108	29
1	3/26/2014	9:05 AM	547.5	1	28.5	7.5	20.5	140	8.28	102	28
1	4/2/2014	9:00 AM	2419.6	1	410.6	18.5	21.6	136	9.57	123	25
1	4/9/2014	9:15 AM	461.1	1	21.1	15	22	136	9.05	124	18
1	4/16/2014	9:05 AM	261.3	1	24.7	12.5	23.4	129	9.23	136	16
1	4/23/2014	9:00 AM	1732.9	1	12.3	21	22.5	175	9.29	171	13
1	4/30/2014	10:00 AM	547.5	1	5.1	27.5	28.2	155	9.72	177	28
1	5/7/2014	9:00 AM	2280	3.1	10.8	23.8	26.7	137	9.1	153	12
1	5/14/2014	9:30 AM	1119.9	1	11	27	28.1	140	10.53	104	30
1	5/15/2014	9:25 AM	224.7	1	4.1	26.5	28.2	123	10.21	98	55
1	5/21/2014	9:00 AM	686.7	1	2	23.6	26.3	107	10.63	92	46
1	5/28/2014	8:56 AM	3990	1	22.6	27	28.8	98	10.34	100	26
1	6/4/2014	9:05 AM	5560	1	185	25.7	27.5	98	9.42	145	18
1	6/11/2014	8:55 AM	2930	1	6.1	24.1	29.7	104	9.37	147	24
1	6/18/2014	9:15 AM	1553.1	1	10.9	27	29.7	100	9.6	162	49
1	6/25/2014	8:55 AM	1553.1	1	21.1	28.7	31	103	9.36	178	25
2	11/7/2012	11:46 AM	9340	7.4	46.5	18.5	21	355	7.98	152	24
2	11/14/2012	11:38 AM	5940	2	124	22.5	21.3	359	8.56	178	17
2	11/21/2012	9:34 AM	8820	7.4	270	18.6	19	368	7.89	173	72
2	11/28/2012	11:39 AM	10810	1	2419.6	25.7	20.8	356	8.88	156	34
2	12/5/2012	11:20 AM	4350	1	524.7	24.6	20.6	332	9.55	155	57
2	12/12/2012	9:40 AM	27550	28.5	2419.6	22.2	22.2	331	9.17	136	58
2	12/19/2012	9:38 AM	22820	1	1203.3	17.9	19.6	360	8.2	145	23
2	12/23/2012	12:20 PM	123600	325.5	2419.6	18.9	17.8	337	7.86	142	35
2	12/26/2012	11:39 AM	34480	30.1	2419.6	23.6	18.1	329	7.97	131	18
2	1/2/2013	11:44 AM	1553.1	1	1553.1	26.3	20.8	319	8.92	168	23
2	1/9/2013	10:11 AM	2419.6	2	231	24.9	20.9	311	9.42	114	35
2	1/16/2013	9:56 AM	1553.1	1	70.3	23.7	21.6	311	9.58	93	39
2	1/23/2013	9:49 AM	2419.6	4.1	1732.9	16.4	17.1	336	8.79	80	16
2	1/30.2013	9:50 AM	8600	3	52.8	23	20.7	334	9	56	14
2	2/6/2013	10:02 AM	2430	1	6.3	22.2	18.3	337	9.28	101	19
2	2/13/2013	10:05 AM	2419.6	1	3.1	23.9	18.6	335	8.97	91	17
2	2/15/2013	10:17 AM	298700	524.7	2880	12.4	18.8	335	8.18	125	26
2	2/17/2013	10:04 AM	61310	156.5	1553.1	9.6	17.1	333	7.96	56	25
2	2/18/2013	10:26 AM	19680	102.2	1986.3	15.7	16.8	340	7.88	144	24
2	2/20/2013	9:53 AM	43600	24.3	172.7	19.4	18.3	334	8.2	147	19
2	2/27/2013	9:55 AM	4140	30.9	1046.2	19.7	21.6	338	8.85	120	21
2	3/6/2013	10:08 AM	2419.6	1	178.2	13.3	17.7	339	8.54	137	29
2	3/13/2013	10:08 AM	2419.6	2	2419.6	14.5	17.8	344	8.78	129	15
2	3/20/2013	9:29 AM	1413.6	2	15.8	21	21.4	349	8.87	117	7
2	3/23/2013	7:46 AM	6488000	11530	15290	17.9	18.7	264	7.7	72	48
2	3/25/2013	10:09 AM	488400	1986.3	2419.6	16.7	19.7	256	7.68	86	12
2	3/27/2013	9:53 AM	86000	238.2	343	11	17.6	275	7.56	23	15
2	4/3/2013	10:11 AM	3500	2	290.4	24.4	22.7	273	9.51	52	30
2	4/6/2013	9:48 AM	36540	53.7	461.1	17.2	20.6	272	9.37	67	37
2	4/8/2013	10:09 AM	4740	1	69.5	26.2	22.3	272	9.7	86	47
2	4/10/2013	10:02 AM	866400	2	31.9	24.6	22.4	279	9.26	100	28
2	4/17/2013	9:26 AM	3450	1	20	23.4	25.2	295	9.17	84	17
2*	4/20/2013	9:53 AM	68670000	9830	8200	28.9	25.2	289	7.84	90	22
2*	4/22/2013	9:42 AM	37840000	14970	3210	20.3	24.1	293	7.44	91	32
2*	4/24/2013	10:13 AM	98040000	2180	2419.6	25.6	24.1	294	7.51	108	29
2	4/30/2013	10:23 AM	816400	435.2	2419.6	23.6	25.4	294	7.67	95	21
2	5/1/2013	10:05 AM	980400	290.3	5450	22.3	25.5	303	7.79	113	20
2	5/2/2013	9:10 AM	24810000	6950	141360	20.2	23.9	299	7.53	68	26
2	5/7/2013	9:59 AM	275500	235.9	2060	20.7	23.8	296	7.34	67	17
2	5/8/2013	9:54 AM	325500	172.2	2419.6	22.2	24.2	298	7.45	64	23
2	5/15/2013	9:54 AM	14670	13.4	90.7	23.1	24.9	305	7.79	14	21
2	5/21/2013	9:59 AM	1119900	727	155310	26.4	25.5	261	7.42	29	56
2	5/22/2013	10:02 AM	920800	365.4	81640	24.4	25.4	262	7.41	18	57
2*	5/24/2013	9:58 AM	579400	461.1	2419.6	26.2	26.9	202	7.19	22	63
2	5/26/2013	9:53 AM	36540	44.1	11190	24.2	25.6	206	7.24	54	46
2	5/29/2013	9:59 AM	8650	6.3	352.4	27.9	26.1	207	7.71	71	34
2	10/9/2013	9:15 AM	50120	25.7	1299.7	22	27.1	210	7.45	75	36
2	10/16/2013	9:30 AM	8860	7.5	261.3	25.6	26	353	7.33	113	26
2	10/23/2013	9:25 AM	16700	16	461.1	24.4	27.3	306	7.86	115	26
2	10/30/2013	9:10 AM	24950	6.3	1203.3	21.5	23.5	315	7.68	86	13
2	11/6/2013	9:15 AM	75560	11	1413.6	25.7	22.8	280	7.46	65	26

2	11/13/2013	9:15 AM	2419.6	6.2	290.9	16.5	22.1	292	7.63	70	28
2	11/20/2013	9:30 AM	241960	3.1	131.7	23.8	22.5	291	7.7	88	26
2	11/27/2013	9:20 AM	14210	7.3	298.7	19.8	21.2	301	7.75	43	39
2	12/3/2013	9:40 AM	2990	1	38.1	21.5	19.2	316	7.77	68	22
2	12/11/2013	9:35 AM	141360	1	49.6	20.2	22	159	8.74	78	27
2	12/18/2013	9:30 AM	20640	12.1	151	12.8	18.4	151	7.41	92	23
2	12/23/2013	9:25 AM	22240	1	68.9	24.2	22.1	171	8.68	80	24
2	12/30/2013	9:20 AM	46740	42.8	3640	16.2	19.6	153	8.12	91	26
2	1/7/2014	9:05 AM	20140	7.5	2419.6	2.3	17	177	7.32	153	21
2	1/8/2014	9:10 AM	16640	5.2	111.7	7	15.6	179	7.37	134	23
2	1/15/2014	9:30 AM	17850	2	20.1	12.9	18	171	7.37	93	28
2	1/22/2014	9:35 AM	155310	14.5	2419.6	7	13.6	175	7.2	142	27
2	1/23/2014	10:05 AM	14550	5.2	113.9	8.6	15.6	181	6.63	120	18
2	1/24/2014	9:30 AM	9090	5.2	67.4	8.6	15.3	179	7.99	149	18
2	1/29/2014	9:10 AM	6770	1	157.6	11.7	18	183	7.2	123	37
2	2/5/2014	9:50 AM	7270	3.1	57.3	23.8	22	171	7.21	178	27
2	2/12/2014	9:30 AM	3590	1	7.2	17.3	18.5	175	7.35	135	20
2	2/13/2014	10:05 AM	198630	285.1	4080	14.9	17.8	158	7.07	79	37
2	2/19/2014	9:18 AM	2010	1	51.2	19	18.2	168	7.79	150	24
2	2/26/2014	9:25 AM	488.4	1	42.5	17.1	21.8	171	9.8	114	37
2	3/5/2014	9:45 AM	1413.6	1	17.7	18.5	21.5	170	9.43	85	31
2*	3/12/2014	9:05 AM	2419.6	1	24.6	20.3	20.9	178	9.13	103	30
2	3/18/2014	9:15 AM	2419600	206.4	17240	16.6	20.2	144	8.44	137	47
2	3/19/2014	9:15 AM	111900	50.4	1553.1	16.1	20.4	147	7.4	127	24
2	3/26/2014	9:15 AM	32550	3.1	2419.6	7.6	19.7	145	8.17	104	35
2	4/2/2014	9:10 AM	21780	19.9	1413.6	18.5	22.1	129	8.46	160	21
2	4/9/2014	9:25 AM	11780	9.7	1732.9	15	21.8	130	8.1	159	20
2	4/16/2014	9:20 AM	3550	12.1	238.7	12.5	23	102	8.57	159	19
2	4/23/2014	9:15 AM	241960	18.3	1986.3	21	22	141	8.27	193	8
2	4/30/2014	10:10 AM	104600	1	34.3	27.6	24.3	143	8.45	166	14
2*	5/7/2014	9:15 AM	1119900	2419.6	29240	23.9	25.3	113	7.6	181	29
2	5/14/2014	9:45 AM	10430	10.9	770.1	26.9	27.1	122	8.21	176	32
2	5/15/2014	9:35 AM	2590	3.1	3360	26.4	27.2	116	8.39	166	29
2	5/21/2014	9:15 AM	12230	6.2	37.2	23.7	24.2	126	8.78	154	37
2	5/28/2014	9:12 AM	54750	1	178.2	27.1	27.4	129	9.35	132	34
2*	6/4/2014	9:20 AM	129920	30.1	2419.6	27.3	26.7	126	7.62	184	32
2	6/11/2014	9:05 AM	111990	4.1	152.9	25	28.7	133	7.41	189	19
2	6/18/2014	9:30 AM	3360	2	110	27.8	28.5	112	7.51	202	32
2	6/25/2014	9:10 AM	19350	5.2	275.5	28.5	29	112	7.59	219	28
3	11/7/2012	12:18 PM	7270	1	68.1	19	21.2	282	6.98	163	20
3	11/14/2012	12:03 PM	11780	1	686.7	22.8	21.8	297	8	206	20
3	11/21/2012	9:58 AM	26030	13.5	66.3	19.9	19.8	305	7.51	180	18
3	11/28/2012	12:04 PM	52470	66.3	41	25.2	20.8	302	8.61	180	20
3	12/5/2012	11:45 AM	141360	1	52.8	26.5	21.4	302	8.69	163	17
3	12/12/2012	10:04 AM	365400	1	307.6	23.1	22.7	304	8.45	155	12
3	12/19.2012	10:00 AM	36540	1	14.6	20.8	20.5	313	7.4	161	22
3	12/23/2012	12:44 PM	34480	7.5	67	20	17.9	312	7.4	160	35
3	12/26/2012	12:02 PM	19180	1	58.3	24.3	18.5	308	7.49	151	21
3	1/2/2013	12:06 PM	2419.6	1	12	26.6	18.4	310	7.82	184	18
3	1/9/2013	10:34 AM	4800	2	23.1	25.7	21	308	8.85	129	15
3	1/16/2013	10:15 AM	2419.6	1	13.4	25.2	22.3	305	9.82	102	27
3	1/23/2013	10:08 AM	1553.1	3	57.6	15.8	17.7	310	8.77	84	16
3	1/30.2013	10:12 AM	11060	11.9	2419.6	25	21	295	9.05	58	16
3	2/6/2013	10:22 AM	5380	1	40.8	21.5	19.1	304	9.78	95	26
3	2/13/2013	10:30 AM	3640	1	35.5	25.4	19	301	9.25	87	82
3	2/15/2013	10:39 AM	4140	1	1413.6	12.4	18.9	297	9.63	116	24
3	2/17/2013	10:26 AM	7760	1	178.5	11.3	17.5	295	9.27	53	27
3	2/18/2013	10:45 AM	8160	1	162.4	15.1	17.5	290	9.23	122	32
3	2/20/2013	10:12 AM	10760	1	141.4	20.8	19.1	288	9.4	131	27
3	2/27/2013	10:17 AM	4800	1	113.7	20.7	21.9	286	9.72	109	46
3	3/6/2013	10:27 AM	2920	1	101.4	19.2	17.6	287	9.12	134	43
3	3/13/2013	10:28 AM	8820	2	185	18.5	18.5	298	8.55	138	48
3	3/20/2013	9:50 AM	2490	1	33.3	20.7	21.3	306	7.97	128	36
3	3/23/2013	8:07 AM	156500	42	2419.6	18	18.8	300	7.36	78	22
3	3/25/2013	10:30 AM	1299700	47.9	980.4	18.8	20.6	292	7.57	91	15
3	3/27/2013	10:13 AM	7490	2	68	12	17.1	295	7.66	28	26
3	4/3/2013	10:45 AM	2490	2	86	25.2	23.8	293	9.94	50	25
3	4/6/2013	10:12 AM	4640	1	48.2	18.3	21.1	285	9.36	62	22
3	4/8/2013	10:31 AM	5650	1	93.4	25.8	22.7	288	9.38	94	19
3	4/10/2013	10:23 AM	8130	1	15.5	24.8	23.1	292	8.88	108	17
3	4/17/2013	9:46 AM	2380	2	42.2	25	25.8	306	8.64	92	16
3	4/20/2013	10:16 AM	209800	5.2	1732.9	19.4	25.4	294	8.58	91	15
3	4/22/2013	10:05 AM	29200	10.9	4570	20.6	24.1	301	8.09	89	17
3	4/24/2013	10:40 AM	24890	1	100.6	25.2	25	299	8.46	109	16
3	4/30/2013	10:51 AM	816400	80.5	8360	26.3	26	283	8.37	96	25

3	5/1/2013	10:26 AM	1732900	56.1	4790	22	25.1	284	7.94	115	23
3	5/2/2013	9:34 AM	1413600	23.3	5280	20.8	23.8	282	7.51	72	24
3	5/7/2013	10:22 AM	547500	1	286.3	23.1	24.8	263	7.51	69	19
3	5/8/2013	10:13 AM	488400	5.2	119.8	23.4	24.5	265	7.67	68	27
3	5/15/2013	10:15 AM	8450	1	97.7	27.7	25.9	271	7.68	7	14
3	5/21/2013	10:21 AM	6440	1	77.1	27.1	28.4	270	8.85	30	19
3	5/22/2013	10:24 AM	16070	1	38.8	25.6	27.7	271	8.58	22	18
3	5/24/2013	10:20 AM	93300	7.4	148.1	29.2	27.8	253	8.61	22	23
3	5/26/2013	10:15 AM	61300	3.1	38.8	24.7	27.1	260	7.78	46	19
3	5/29/2013	10:20 AM	26130	3	98.3	26.3	26.8	268	7.58	52	18
3	10/9/2013	9:45 AM	16700	7.4	1046.2	21.2	27.8	249	7.62	76	28
3	10/16/2013	10:00 AM	10460	24.1	727	24.7	27.2	281	7.01	95	16
3	10/23/2013	9:45 AM	27230	90.8	770.1	23	27.2	297	7.06	112	30
3	10/30/2013	9:30 AM	6970	28.5	579.4	21.2	23.8	322	7.47	98	20
3	11/6/2013	9:40 AM	10360	6.3	146.7	25	24.2	320	7.32	53	26
3	11/13/2013	9:35 AM	2419.6	2	184.2	16.6	22.1	320	7.5	72	33
3	11/20/2013	9:50 AM	241960	10.7	686.7	24	22.9	313	7.62	85	22
3	11/27/2013	9:40 AM	68670	3	114.5	19.1	21.6	321	7.45	44	27
3	12/3/2013	10:00 AM	14550	3.1	80.9	21.7	19.6	313	7.84	70	24
3	12/11/2013	10:05 AM	7710	10.9	24.6	21.4	22.4	172	8.68	63	22
3	12/18/2013	10:00 AM	2930	9.6	52.9	14.6	19	170	7.18	90	40
3	12/23/2013	9:50 AM	4730	8.5	191.8	25.3	22.2	177	7.9	93	26
3*	12/30/2013	9:40 AM	19890	39.7	396.8	15.9	19.7	163	5.11	91	34
3	1/7/2014	9:25 AM	4070	3.1	48.8	2.8	16	167	7.37	157	17
3	1/8/2014	9:35 AM	5250	8.4	51.2	7.2	14.1	168	7.33	136	21
3*	1/15/2014	10:10 AM	9080	13.1	4.1	13.1	18.1	163	7.35	101	23
3	1/22/2014	10:00 AM	2419.6	5	261.3	8.4	15.4	159	7.07	140	29
3	1/23/2014	10:30 AM	2490	1	23.3	8.7	15	147	6.78	125	22
3	1/24/2014	9:55 AM	2280	1	14.4	9.1	14.1	156	6.91	141	23
3	1/29/2014	9:30 AM	2419.6	3	37.3	12.6	27.7	161	7.09	124	22
3	2/5/2014	10:15 AM	3890	1	82.1	25.6	22	151	7.12	148	22
3	2/12/2014	9:45 AM	111990	1	6.3	17.7	18.8	157	7.36	137	15
3	2/13/2014	10:25 AM	241960	46.7	2419.6	17	18.5	145	6.98	91	38
3	2/19/2014	9:43 AM	5460	2	2419.6	20.8	19	150	7.93	119	27
3	2/26/2014	9:50 AM	198630	2	7.3	21	22.5	156	9.7	122	12
3	3/5/2014	10:10 AM	15220	14.2	10.7	20.1	21.8	153	9.07	109	9
3	3/12/2014	9:25 AM	9870	1	5.1	20.3	21.5	154	9.14	98	10
3	3/18/2014	9:30 AM	198630	7.4	3990	16.7	21	145	8.9	107	33
3	3/19/2014	9:35 AM	241960	10.9	1299.7	16.4	21.2	144	8.41	94	18
3	3/26/2014		19350	2	111.9	8	20.7	143	7.7	122	17
3	4/2/2014	9:30 AM	241960	2	517.2	22.5	21.7	139	9.18	130	9
3	4/9/2014	9:45 AM	241960	2	2460	15.9	22.7	142	8.16	146	6
3	4/16/2014	9:40 AM	14390	1	461.1	13	23.6	145	8.06	194	15
3	4/23/2014	9:35 AM	241960	1	240	21	22.8	145	8.13	199	2
3	4/30/2014	10:40 AM	15650	1	9.4	27.7	27.8	148	9.48	133	2
3	5/7/2014	9:35 AM	92080	1	416	24.5	27.1	125	8.75	155	14
3	5/14/2014	10:00 AM	193500	1	547.5	28	27.7	131	8.1	180	8
3	5/15/2014	10:00 AM	13540	1	1553.1	29.5	27.7	121	8.14	170	22
3	5/21/2014	9:35 AM	2330	1	13.2	25.1	26.4	128	8.16	162	21
3	5/28/2014	9:33 AM	25950	1	82	28	28.6	130	8.96	143	16
3	6/4/2014	9:45 AM	7590	3.1	204.6	27.8	27	128	7.53	185	20
3	6/11/2014	9:25 AM	7120	5.2	40.4	25.1	29	134	7.17	194	9
3	6/18/2014	9:45 AM	5190	1	137.6	28.3	29.6	128	7.12	201	16
3	6/25/2014	9:35 AM	7710	1	113.9	30.6	30.3	131	7.2	223	9
4	11/7/2012	12:37 PM	13540	76.3	613.1	18	21.1	397	9.04	134	76
4	11/14/2012	12:17 PM	6200	67	325.5	22.7	22	396	9.17	163	96
4	11/21/2012	10:16 AM	9850	95.9	387.2	20.5	19.2	424	7.97	178	55
4	11/28/2012	12:17 PM	6890	41.4	365.4	24.9	20.7	411	9.07	177	85
4	12/5/2012	11:58 AM	129970	70.8	161.6	26.3	21.1	418	8.77	175	74
4	12/12/2012	10:18 AM	43520	167	770.1	24.4	23.5	435	7.43	173	52
4	12/19/2012	10:15 AM	83600	135.4	365.4	17.6	20.1	439	7.95	169	69
4*	12/23/2012	12:57 PM	26130	235	1046.2	20.2	15.9	423	8.91	158	89
4*	12/26/2012	12:14 PM	10460	365.4	2920	24.4	18.7	413	9.25	145	77
4	1/2/2013	12:22 PM	8010	104.6	307.6	26.6	21.8	401	9.47	162	59
4	1/9/2013	10:47 AM	5540	29.2	193.5	25.2	19.9	404	9.16	144	71
4	1/16/2013	10:28 AM	10120	27.5	613.1	26.3	22.5	412	8.63	127	43
4	1/23/2013	10:20 AM	5710	14.5	142.1	15.4	16.6	411	8.68	88	41
4	1/30.2013	10:23 AM	12670	7.4	128.1	25.5	21.1	408	9.17	68	52
4	2/6/2013	10:34 AM	30760	1	139.6	24.2	18.1	393	9.75	104	51
4	2/13/2013	10:43 AM	14390	12.1	75.2	25.8	15.6	386	9.08	100	44
4*	2/15/2013	10:51 AM	24890	24.3	686.7	13.1	19.1	396	8.18	130	30
4	2/17/2013	10:43 AM	3970	5.2	67.6	8.7	15.6	394	8.45	63	33
4	2/18/2013	10:58 AM	21870	9.8	378.4	16.3	15.6	387	8.46	134	41
4	2/20/2013	10:24 AM	3730	1	21.8	20.6	18.8	379	8.77	140	32
4	2/27/2013	10:32 AM	51720	56.1	184.2	20.8	22.5	389	8.84	123	47

4	3/6/2013	10:41 AM	4500	20.3	95.8	15.1	17.6	381	9.09	138	61
4	3/13/2013	10:41 AM	20980	9.7	410.6	17.5	18.3	387	9.21	133	72
4	3/20/2013	10:02 AM	23590	5.2	307.6	22.1	21.5	382	9.1	125	44
4	3/23/2013	8:20 AM	248100	517.2	5200	18.3	18.9	367	8.83	72	48
4	3/25/2013	10:41 AM	866400	83.3	2419.6	18.5	21.3	365	7.96	94	30
4	3/27/2013	10:22 AM	43200	6.3	2419.6	10.2	17	370	8.77	30	38
4	4/3/2013	10:59 AM	76300	4.1	2419.6	25.6	23	372	9.05	38	21
4	4/6/2013	10:28 AM	41060	12.2	2419.6	18.4	20.8	367	8.99	55	37
4	4/8/2013	10:45 AM	9880	1	816.4	25.8	22.4	363	9.38	95	38
4	4/10/2013	10:26 AM	8600	1	236.7	26	22.7	358	9.31	106	58
4	4/17/2013	9:59 AM	17260	17.1	2419.6	26.1	25.8	365	9.24	89	76
4	4/20/2013	10:30 AM	37840	7.5	2419.6	18.4	25	354	8.76	92	68
4	4/22/2013	10:20 AM	85700	178.5	2419.6	21.6	23.8	372	7.92	92	61
4	4/24/2013	10:53 AM	5120	3.1	1732.9	26.9	24.7	373	7.84	118	57
4	4/30/2013	11:06 AM	365400	298.7	6440	26.9	26.2	358	8.3	99	70
4	5/1/2013	10:39 AM	231000	360.9	2280	22	25.2	360	8.35	117	72
4*	5/2/2013	9:51 AM	387300	770.1	29090	21.1	23.8	353	8.05	73	74
4	5/7/2013	10:37 AM	17250	18.9	2419.6	22.9	24.2	311	8.86	65	78
4	5/8/2013	10:26 AM	5120	12	201.4	22.9	25.2	311	9.28	80	87
4	5/15/2013	10:28 AM	36540	1	231	27.2	25.7	333	8.25	5	40
4	5/21/2013	10:37 AM	26030	23.1	1986.3	27.5	27.2	332	8.67	21	46
4	5/22/2013	10:40 AM	10760	8.6	290.9	29.6	27.1	338	8.32	32	48
4	5/24/2013	10:34 AM	15150	17.1	360.9	27.8	28.6	326	8.91	14	54
4	5/26/2013	10:30 AM	22470	9.8	488.4	25.5	27.2	340	8.3	41	40
4	5/29/2013	10:34 AM	16700	10.8	1119.9	26.9	26.8	338	8.37	46	49
4	10/9/2013	10:00 AM	17260	1	260.3	22.9	27.2	204	7.98	76	40
4	10/16/2013	10:10 AM	17230	1	224.7	24.6	26.1	187	8.9	70	51
4	10/23/2013	10:00 AM	34480	23.8	2419.6	24.5	27.1	217	7.67	107	48
4	10/30/2013	9:40 AM	17230	8.5	488.4	22.5	22.9	272	8.23	88	61
4	11/6/2013	9:50 AM	38730	28.1	2419.6	26.1	22.9	267	7.95	49	53
4	11/13/2013	9:50 AM	2419.6	8.5	686.7	16.5	21.1	277	8.64	60	57
4	11/20/2013	10:00 AM	26130	122.2	1553.1	24	22.8	288	8.36	80	67
4	11/27/2013	9:55 AM	13340	42.6	686.7	19.6	21.2	290	8.45	38	76
4	12/3/2013	10:15 AM	7890	3	648.8	21.6	19.5	294	8.59	63	73
4	12/11/2013	10:15 AM	10860	2	517.2	21.6	22.4	159	8.89	61	90
4	12/18/2013	10:05 AM	11530	2	248.1	14.6	17.9	157	8.9	87	70
4	12/23/2013	10:00 AM	11980	1	410.6	24.9	21.9	172	9.13	92	51
4	12/30/2013	9:50 AM	19040	56.9	1732.9	16.1	19.6	160	5.09	88	60
4	1/7/2014	9:40 AM	21780	29.2	1119.9	4.5	14.7	169	7.29	149	40
4	1/8/2014		12670	30.5	1553.1	7.8	13.2	168	7.3	127	43
4*	1/15/2014	10:10 AM	3640	1	122.3	13.3	17.9	167	7.33	8.3	54
4	1/22/2014	10:10 AM	6090	7.4	290.9	8.5	15	160	7.09	131	39
4	1/23/2014	10:45 AM	2280	7.4	89.2	9.8	14.3	156	6.69	102	37
4	1/24/2014	10:05 AM	1985.3	17.3	63.3	9.2	13.3	157	6.84	124	38
4	1/29/2014	9:40 AM	16160	3	10080	12.1	17.3	162	6.55	103	46
4	2/5/2014	10:30 AM	12590	4.1	1299.7	24.8	21.6	152	7.13	142	39
4	2/12/2014	10:00 AM	2750	3	57.1	19	19.2	163	7.26	110	35
4	2/13/2014	10:30 AM	15610	154.1	2419.6	15.2	18.6	149	7.07	51	50
4	2/19/2014		14210	1	34.5	18.8	18.8	153	8.27	110	47
4*	2/26/2014	10:00 AM	33100	28.8	547.5	19.1	22.2	172	8.94	154	98
4	3/5/2014	10:20 AM	241960	4.1	613.1	20.4	22.5	168	8.8	121	118
4*	3/12/2014	9:40 AM	28090	22.6	2419.6	20.8	21.5	166	8.86	103	77
4	3/18/2014	9:45 AM	162400	2419.6	10980	17	20.6	119	8.64	123	129
4*	3/19/2014	9:45 AM	241960	1732.9	2419.6	17.2	21.5	121	7.99	114	65
4	3/26/2014	9:50 AM	73.8	1	106.7	7.8	19.1	126	9.17	82	74
4	4/2/2014	9:40 AM	3840	18.9	195.6	20.8	21.2	110	10.37	83	53
4	4/9/2014	10:00 AM	3990	2	488.4	15.8	22.1	113	10.07	90	62
4	4/16/2014	9:50 AM	2650	5.1	153.9	13.5	22.6	115	9.59	126	71
4	4/23/2014	9:40 AM	11220	7.4	172.3	21	22.6	126	9.83	143	50
4	4/30/2014	10:50 AM	32550	9.7	1119.9	27.8	27.6	137	8.53	162	31
4	5/7/2014	9:40 AM	61310	10.9	1732.9	24.4	26.9	125	8.3	163	43
4	5/14/2014	10:10 AM	241960	7.4	290.9	28.1	27.8	134	8.32	172	28
4	5/15/2014	10:10 AM	72700	9.8	1553.1	28.9	28.1	117	8.17	172	33
4	5/21/2014	9:45 AM	81640	2	57.6	25	26	130	8.91	140	44
4	5/28/2014	9:44 AM	23820	1	248.1	27.8	28.1	135	9.36	125	21
4	6/4/2014	9:55 AM	14010	1	2419.6	28.4	27.1	130	9.25	144	58
4	6/11/2014	9:35 AM	12590	5.2	191.8	26.9	29	140	8.56	151	36
4	6/18/2014	9:55 AM	11260	10.9	195.1	28.5	29.4	128	7.59	184	37
4	6/25/2014	9:40 AM	17250	2	13.5	30.5	30.5	135	7.89	195	38
5	11/7/2012	12:53 PM	9600	43.2	204.6	19.9	22.3	265	7.14	188	52
5	11/14/2012	12:32 PM	8620	3.1	517.2	24.7	22.4	273	8.63	178	31
5	11/21/2012	10:27 AM	7890	11	101.9	23	20.5	276	8.16	177	23
5	11/28/2012	12:25 PM	13340	15.3	365.4	26.3	23.1	270	9.49	169	44
5	12/5/2012	12:10 PM	11120	25.6	38.8	29.3	23	271	9.21	159	38
5	12/12/2012	10:36 AM	5810	31.8	228.2	27.5	23.6	272	8.59	175	18

5	12/19/2012	10:25 AM	11060	191.8	1299.7	22.3	21	286	7.41	164	11
5	12/23/2012	1:06 PM	22240	201.4	461.1	19.5	18.4	283	7.35	169	20
5	12/26/2012	12:22 PM	10760	209.8	235.9	23.1	19.8	278	7.71	165	17
5	1/2/2013	12:33 PM	3550	96	31.3	26	20.8	275	7.94	175	8
5	1/9/2013	10:56 AM	5910	980.4	218.7	25.8	21.7	276	7.77	157	5
5	1/16/2013	10:37 AM	2430	32.7	8.5	26.6	22.7	268	9.56	124	8
5	1/23/2013	10:30 AM	19680	686.7	123.6	18.4	18.3	267	8.86	86	10
5	1/30.2013	10:33 AM	8290	62.4	98.8	25.7	22.4	268	9.69	61	23
5	2/6/2013	10:42 AM	2419.6	37.9	248.1	23.4	19.7	271	10.58	82	27
5	2/13/2013	10:53 AM	2750	4.1	19.7	26.2	19.4	322	9.68	102	20
5	2/15/2013	11:08 AM	14670	83.6	139.6	15.5	19.1	251	9.32	117	20
5	2/17/2013	10:55 AM	7710	38.8	72.7	12.6	18.4	260	9.38	58	20
5	2/18/2013	11:08 AM	2920	15.8	33.1	19.5	18.7	254	9.32	113	25
5	2/20/2013	10:32 AM	2419.6	18.7	43.1	25.1	20.4	252	9.89	115	30
5	2/27/2013	10:43 AM	1413.6	3.1	11	24.7	22.6	250	9.98	115	26
5	3/6/2013	10:49 AM	228.2	6.3	16	16.2	29	259	10.25	112	36
5	3/13/2013	10:50 AM	613.1	32.3	71.7	19.4	18.4	252	10.14	110	51
5	3/20/2013	10:12 AM	135.4	7.5	62.4	20.7	22	267	10.48	100	37
5	3/23/2013	8:30 AM	866.4	12.1	2419.6	19	19.3	225	9.97	61	45
5	3/25/2013	10:49 AM	4200	2	2419.6	20.9	21.5	207	9.5	81	37
5	3/27/2013	10:30 AM	4500	11	54.4	13.3	17.9	215	9.72	21	42
5	4/3/2013	11:11 AM	1732.9	21.6	44.8	27.7	23.8	231	7.91	28	5
5	4/6/2013	10:41 AM	83900	4.1	26.2	20.8	22	232	7.78	44	4
5	4/8/2013	11:01 AM	435200	16.1	48.2	25.9	23.6	236	8.32	105	7
5	4/10/2013	10:45 AM	9080	2	34.5	27.5	24.1	232	9.72	100	13
5	4/17/2013	10:09 AM	3930	2	40.2	27.4	26.2	233	9.78	84	14
5	4/20/2013	10:42 AM	2419.6	43.5	117.8	19.5	25.8	222	10.03	80	17
5	4/22/2013	10:23 AM	1046.2	1	13.2	21.5	24.2	227	9.51	79	15
5	4/24/2013	11:06 AM	5730	1	28.7	26.3	25.5	228	10.16	90	54
5	4/30/2013	11:18 AM	16160	36.4	5560	27	28	213	10.18	77	39
5	5/1/2013	10:49 AM	5290	151	1046.2	22.1	25.1	206	9.86	98	28
5	5/2/2013	10:04 AM	185000	290.9	18500	22.4	23.8	204	8.66	66	18
5	5/7/2013	10:49 AM	7940	2	629.4	23.6	25.2	176	9.67	59	25
5	5/8/2013	10:36 AM	2419.6	7.5	146.7	27.5	25.7	181	9.77	49	27
5	5/15/2013	10:36 AM	3450	1	27.2	27.2	26.6	191	10.05	25	18
5	5/21/2013	10:49 AM	2880	1	109.5	28.4	28.9	191	10.06	30	22
5	5/22/2013	10:49 AM	69100	2	48.1	26.5	29	188	9.87	17	18
5	5/24/2013	10:45 AM	2419.6	1	13.4	29.5	28.6	188	9.84	19	16
5	5/26/2013	10:40 AM	13140	2	196.8	26.2	28	192	9.45	43	21
5	5/29/2013	10:44 AM	14830	5.2	686.7	25.3	27.6	190	9.6	49	18
5	10/9/2013	10:10 AM	29240	6.3	2419.6	21.3	27.8	169	8.02	74	27
5	10/16/2013	10:20 AM	29240	5.2	2419.6	24	27.2	260	8.03	68	26
5	10/23/2013	10:15 AM	7120	5.2	261.3	24.2	27.2	271	9.03	93	37
5	10/30/2013	9:45 AM	3010	1	110	21.1	23.3	283	8.62	61	31
5	11/6/2013	10:00 AM	9060	24.1	285.1	24	23.6	225	7.61	64	26
5	11/13/2013	10:00 AM	3590	15.8	307.6	17.8	22.1	263	8.96	52	30
5	11/20/2013	10:15 AM	8570	66.3	101.2	24.4	22.8	262	7.41	76	14
5	11/27/2013	10:10 AM	9590	284.2	111.2	19.3	21.8	265	9.01	35	22
5	12/3/2013	10:20 AM	4100	14.6	17.5	22	19.6	255	9.39	53	18
5	12/11/2013	10:45 AM	19680	21.8	184.2	17.8	22.1	160	8.5	136	14
5	12/18/2013	10:15 AM	19350	36.4	148.3	14.4	19.2	137	8.7	86	24
5	12/23/2013	10:15 AM	6770	3.1	115.3	25	22.9	144	9.54	84	75
5	12/30/2013	10:00 AM	3360	14.1	579.4	16.3	19.8	121	5.08	85	59
5	1/7/2014	9:50 AM	8550	4.1	222.4	4.2	16.7	134	7.28	143	53
5	1/8/2014	10:00 AM	6310	7.5	325.5	8.2	14.8	128	7.25	124	52
5	1/15/2014	10:30 AM	2419.6	5.2	10.6	13.2	18	149	7.4	82	51
5	1/22/2014	10:15 AM	2419.6	2	66.3	8.5	16.7	134	6.96	125	41
5	1/23/2014	10:50 AM	2419.6	1	30.1	9.8	16.2	126	6.61	133	36
5	1/24/2014	10:10 AM	2419.6	3	23.8	9.5	15	129	6.79	123	31
5	1/29/2014	9:50 AM	8570	2	32.9	12.2	17.4	133	6.6	130	16
5	2/5/2014	10:35 AM	12340	1	24.1	24.8	22	136	7.09	126	13
5	2/12/2014	10:05 AM	325.5	1	6.1	19.1	19.2	132	7.24	80	15
5	2/13/2014		1203.3	1	365.4	16.1	19.6	123	6.98	22	25
5	2/19/2014		816.4	1	18.3	19.9	20.1	129	8.23	131	30
5*	2/26/2014	10:10 AM	1299.7	1	4.1	19.1	23	135	10.28	89	29
5	3/5/2014	10:30 AM	123600	1	15.5	22.2	22.8	128	9.79	82	21
5	3/12/2014	9:50 AM	19890	1	23.5	20.8	21.5	130	9.63	75	31
5	3/18/2014	9:50 AM	41060	21.1	6500	16.9	21.4	106	9.54	82	47
5*	3/19/2014	10:00 AM	26130	50.4	2419.6	18.1	21.7	105	8.66	83	31
5	3/26/2014	9:50 AM	22.3	1	62.9	8.5	21.2	109	8.57	107	27
5	4/2/2014	9:45 AM	46110	19.7	261.3	20.7	21.8	87	9.72	108	25
5	4/9/2014	10:05 AM	13140	5.2	613.1	16.1	23.4	90	8.81	130	10
5	4/16/2014	10:00 AM	2010	2	47.9	13.5	23.6	90	8.61	162	22
5	4/23/2014	9:50 AM	3090	1	111.8	21.2	22.9	95	10.51	113	45
5	4/30/2014	10:55 AM	241960	2	462.4	27.8	27.9	93	10.71	83	26

5	5/7/2014	9:50 AM	22820	22.6	648.8	24.7	27.1	83	9.38	136	27
5	5/14/2014	10:15 AM	18600	3.1	461.1	28	27.8	87	10.05	122	57
5	5/15/2014	10:15 AM	20640	14.6	2490	28.5	28.2	7.3	9.33	143	69
5	5/21/2014	9:55 AM	9870	15.6	49	25.1	26.1	84	9.42	127	43
5	5/28/2014	9:51 AM	4350	1	201.4	28.3	28.1	87	10.44	83	45
5	6/4/2014	10:05 AM	2690	2.1	1732.9	28.4	27.4	81	9.6	132	73
5	6/11/2014	9:40 AM	68670	70.8	131.4	27.1	28.3	87	9.14	132	54
5	6/18/2014	10:05 AM	12590	8.5	313	28.4	29.5	60	8.91	159	54
5	6/25/2014	9:45 AM	4500	2	8.6	30.6	30.7	69	9.69	142	45
6	11/7/2012	1:19 PM	17850	8.6	547.5	20.8	22	329	7.25	162	31
6	11/14/2012	12:49 PM	20640	160.7	866.4	23.7	21.3	344	7.14	200	25
6	11/21/2012	10:50 AM	20980	2	613.1	23.9	19.7	347	7.34	184	24
6	11/28/2012	12:45 PM	12590	1	146.7	27	20.2	347	7.14	191	32
6	12/5/2012	12:24 PM	6370	1	93.4	30.1	23.8	345	7.23	178	42
6	12/12/2012	10:48 AM	9320	1	82.9	29.1	24.3	347	7.12	178	13
6	12/19/2012	10:41 AM	3310	1	20.6	23.4	21.8	355	7.28	169	13
6	12/23/2012	1:19 PM	157600	98.8	2419.6	21.8	19.2	341	7.13	173	25
6	12/26/2012	12:33 PM	3990	2	343.6	20.6	19.3	333	7.58	164	17
6	1/2/2013	12:50 PM	4870	1	47.5	26.3	23.5	328	8.88	167	26
6	1/9/2013	11:16 AM	2419.6	3.1	19.9	25.7	22.7	322	8.05	164	22
6	1/16/2013	10:48 AM	3590	1	27.5	26.2	23.7	349	7.7	149	17
6	1/23/2013	10:41 AM	2130	2	48	19	18.3	252	8.55	98	14
6	1/30.2013	10:45 AM	2010	1	34.1	25.5	23	342	9.12	75	19
6	2/6/2012	10:54 AM	648800	1	34.1	22.4	21	339	9.87	113	11
6	2/13/2013	11:05 AM	198630	5.2	6.3	26.2	20.9	273	8.92	81	32
6	2/15/2013	11:21 AM	142100	5.2	325.5	14.9	20.7	327	7.97	135	24
6	2/17/2013	11:07 AM	52900	1	160.7	13.3	19.7	329	8.2	70	17
6	2/18/2013	11:20 AM	54750	3.1	183.5	17	18.5	320	8.11	130	24
6	2/20/2013	10:44 AM	26130	1	101.4	21.6	19.9	306	8.78	135	15
6	2/27/2013	10:56 AM	2280	1	38.4	23	23.1	285	8.8	127	13
6	3/6/2013	11:01 AM	4200	1	98.4	18.5	19.6	304	8.57	132	32
6	3/13/2013	11:03 AM	17890	4.1	1046.2	20.5	20	296	8.82	129	31
6	3/20/2013	10:24 AM	6130	2	139.1	22.3	23.3	294	9.12	111	60
6	3/23/2013	8:45 AM	8664000	2720	92080	18.7	21	282	8.24	78	43
6	3/25/2013	11:00 AM	143900	135.1	1732.9	19	22.1	267	8.12	94	15
6	3/27/2013	10:42 AM	54600	26.9	157	13.6	18.9	260	8.98	31	29
6	4/3/2013	11:23 AM	8160	3	42.8	27.9	24	266	9.55	30	36
6	4/6/2013	10:41 AM	613100	61.3	3270	20.9	22.4	315	7.55	35	19
6	4/8/2013	11:01 AM	51200	8.4	2419.6	30.5	24.6	326	7.71	114	17
6	4/10/2013	10:45 AM	17890	5.2	163.8	26.9	25.1	314	8.88	112	36
6	4/17/2013	10:09 AM	6700	1	34.1	26.9	25.9	318	9.15	92	19
6	4/20/2013	10:42 AM	23590	4.1	316.9	20.4	25.4	310	8.56	95	25
6	4/22/2013	10:23 AM	4800	5.2	101	21.5	23.5	317	8.07	93	28
6	4/24/2013	11:06 AM	2419.6	5.2	191.8	28.3	26.3	304	9.35	93	81
6	4/30/2013	11:18 AM	107600	225.4	2419.6	27.5	27.7	301	9.01	88	25
6	5/1/2013	10:49 AM	54750	67	613.1	21.8	25.2	300	9.23	106	22
6	5/2/2013	10:04 AM	93300	44.3	2419.6	22.9	24	311	8.66	71	17
6	5/7/2013	10:49 AM	155310	70.3	2419.6	22.2	25	272	9.3	65	37
6	5/8/2013	10:36 AM	9580	24.6	1732.9	27	25.4	280	9.32	62	28
6	5/15/2013	10:36 AM	60500	128.1	2419.6	27.8	26.3	310	8.96	8	30
6	5/21/2013	10:49 AM	161600	260.3	2419.6	27.6	27.9	314	8.72	10	21
6	5/22/2013	10:49 AM	83300	259.5	2419.6	27.5	29.7	312	8.8	33	28
6	5/24/2013	10:45 AM	344800	133.6	579.4	29	28.7	301	8.37	2	23
6	5/26/2013	10:40 AM	86640	135.4	1299.7	27.2	26.8	307	8.7	34	18
6	5/29/2013	11:01 AM	137600	275.5	2419.6	26.5	26.9	312	8.81	36	27
6	10/9/2013	10:25 AM	173290	55.2	2419.6	21.6	26.2	271	7.68	73	107
6	10/16/2013	10:30 AM	29090	12.2	2810	25.6	26.7	324	7.14	69	49
6	10/23/2013	10:30 AM	21420	69.7	2419.6	22.7	26.8	28	7.17	103	81
6	10/30/2013	10:00 AM	9590	9.7	178.2	24.5	23.2	330	7.39	80	20
6	11/6/2013	10:15 AM	10500	26.9	365.4	28	23.8	281	7.07	58	30
6	11/13/2013	10:15 AM	12460	2	260.3	19.9	22.2	312	7.29	70	46
6	11/20/2013	10:30 AM	10190	4.1	172.3	24.9	23.1	312	6.7	73	34
6	11/27/2013	10:15 AM	9330	10.7	139.6	19	21.4	399	6.94	40	38
6	12/3/2013	10:30 AM	9850	2	107.1	23	19.8	326	6.66	74	37
6	12/11/2013	10:30 AM	20460	6.3	145.5	21.7	22.4	189	7.2	75	39
6	12/18/2013	10:30 AM	17850	2	344.8	14.9	18.2	137	8.68	85	36
6	12/23/2013	10:25 AM	30760	3.1	365.4	25	23.3	188	7.13	117	
6	12/30/2013	10:20 AM	167000	111.2	9330	15.6	20	176	5.37	90	62
6	1/7/2014	10:00 AM	22090	17.1	770.1	4.2	17.1	183	7.23	157	24
6	1/8/2014	10:10 AM	7760	5.1	181.9	9.9	14.5	171	7.3	134	20
6	1/15/2014	10:30 AM	24890	13.2	29.7	18.1	13.4	190	7.35	40	24
6	1/22/2014	10:30 AM	7490	14.6	1732.9	8.6	17.9	178	7.03	144	37
6	1/23/2014	11:10 AM	11520	5.2	410.6	10.3	16.1	173	6.61	149	44
6	1/24/2014	10:20 AM	12960	5.2	125.9	10.3	17.3	183	6.68	143	33
6	1/29/2014	10:00 AM	5200	3.1	1222.2	12.2	19.1	180	6.95	139	32

6	2/5/2014	10:50 AM	4020	7.5	38.6	25.3	23	174	7.03	185	19
6	2/12/2014	10:20 AM	6050	1	16	19.2	21.2	172	7.3	168	17
6	2/13/2014	10:50 AM	48840	141.4	2419.6	15	20.5	163	7.06	107	43
6	2/19/2014	10:15 AM	77.6	1	36.7	22.1	21.1	176	7.87	140	48
6	2/26/2014	10:25 AM	13960	1	9.6	19.1	23.7	177	8.74	169	6
6	3/5/2014	10:45 AM	9850	1	64.5	22.3	22.9	168	8.91	122	32
6	3/12/2014	9:55 AM	10810	1	31.8	20.8	21.8	171	9.03	95	12
6	3/18/2014	10:05 AM	1553100	550.4	104620	17	21.8	156	8.05	153	55
6*	3/19/2014	10:10 AM	201400	61.4	4480	17.9	21.9	153	7.33	134	24
6	3/26/2014	10:05 AM	241960	6.3	1986.3	9	20.8	168	7.87	137	28
6	4/2/2014	10:00 AM	98800	20.3	2419.6	21.8	22.3	148	8.02	164	29
6	4/9/2014	10:15 AM	95600	3.1	122.3	16.2	23.4	162	7.77	162	15
6	4/16/2014	10:10 AM	129970	3.1	120.1	13.9	23.1	168	8.07	193	32
6	4/23/2014	10:00 AM	7170	3.1	40.5	21.2	23.1	167	9.47	155	24
6	4/30/2014	11:05 AM	272300	26.9	549.3	28	28.3	165	9.5	135	30
6	5/7/2014	10:00 AM	866400	3.1	104.6	25.3	27.3	156	9.48	135	25
6*	5/14/2014	10:30 AM	14550	20.3	365.4	28.5	28.1	169	9.79	134	27
6*	5/15/2014	10:25 AM	13740	13.5	461.1	28.4	28.2	154	9.55	133	48
6	5/21/2014	10:00 AM	18420	46.7	344.8	25.2	26.4	176	8.24	167	30
6	5/28/2014	10:04 AM	8160	1	162.4	27.6	27.2	161	9.26	127	29
6	6/4/2014	10:15 AM	13340	5.2	920.8	28.5	27.5	173	8.14	173	22
6	6/11/2014	9:50 AM	7580	2	95.9	26.3	27.6	168	8.32	162	28
6	6/18/2014	10:15 AM	22820	77.1	145	28.5	29.6	116	7.76	189	27
6	6/25/2014	10:00 AM	12590	2	8.3	30.6	31	137	7.81	199	8

* Samples tested positive for *Salmonella* .
