# Supplementary figures and images for: Microbial quality of agricultural water in Central Florida
Source: PLoS One. 2017 Apr 11;12(4):e0174889. doi: 10.1371/journal.pone.0174889 (PMC5388333; doi:10.1371/journal.pone.0174889)

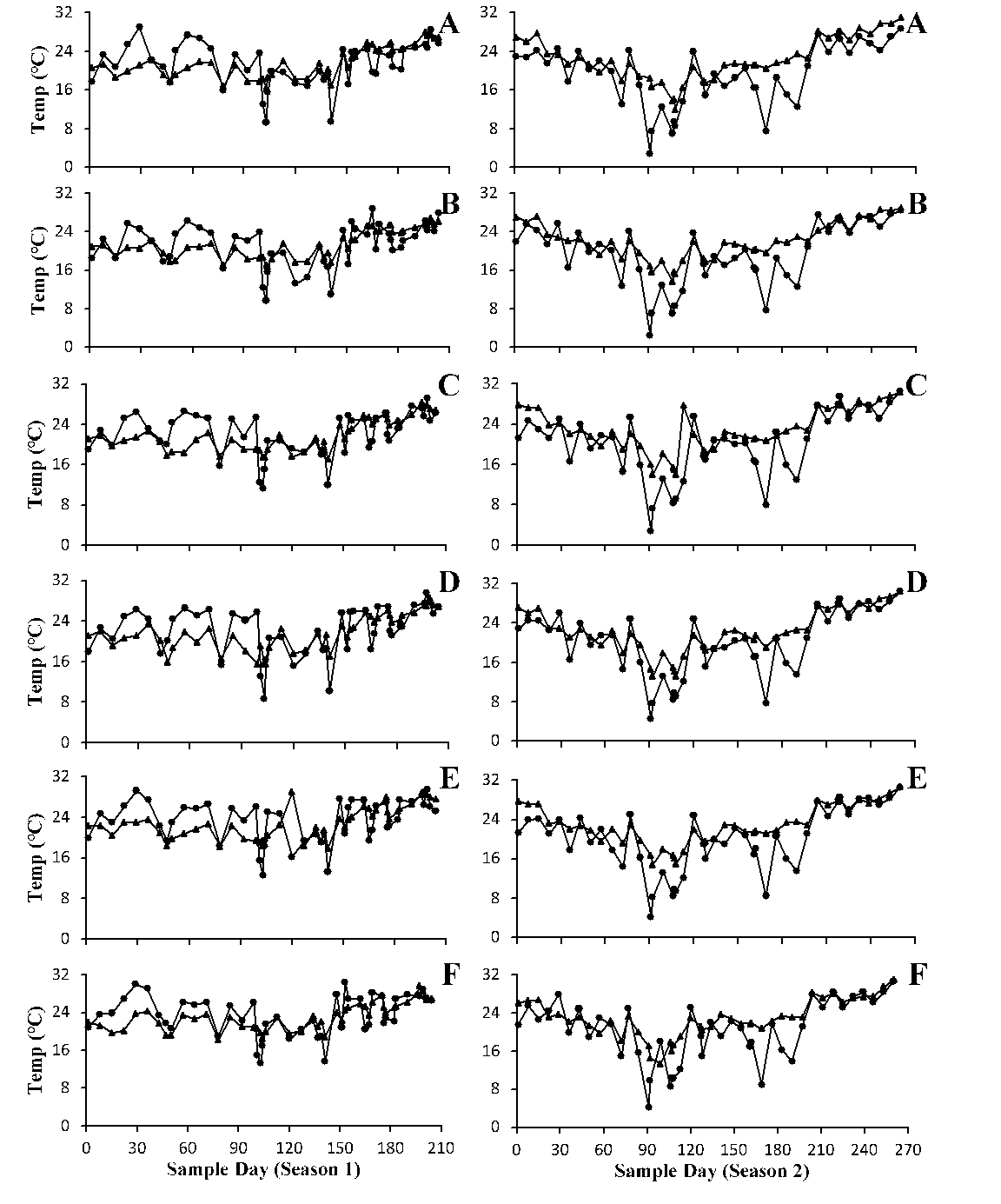

Supplement: S1 Fig — (TIF) [file pone.0174889.s002.tif]

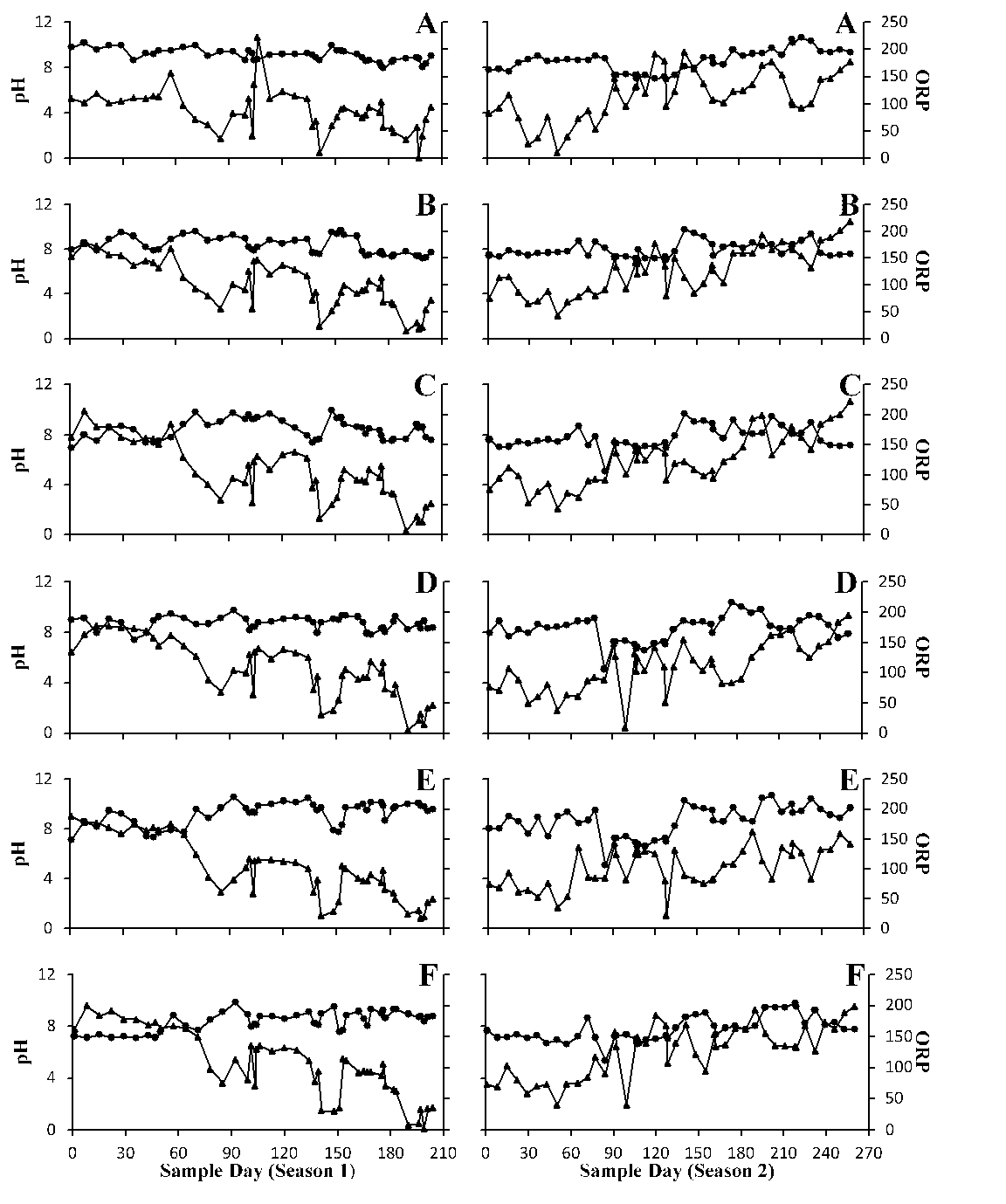

Supplement: S2 Fig — (TIF) [file pone.0174889.s003.tif]

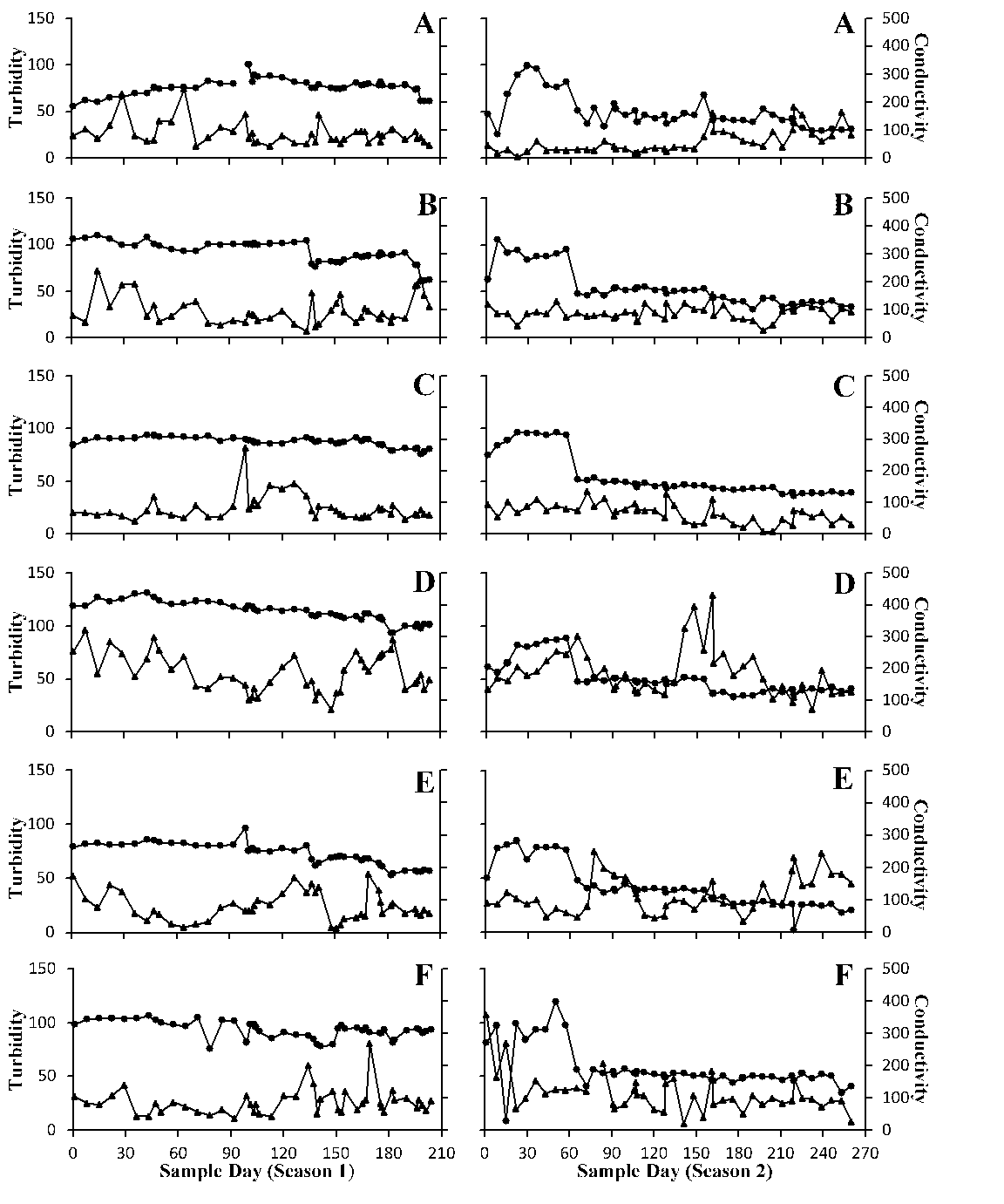

Supplement: S3 Fig — (TIF) [file pone.0174889.s004.tif]
